# Mutation Frequency and Spectrum of Mutations Vary at Different Chromosomal Positions of *Pseudomonas putida*

**DOI:** 10.1371/journal.pone.0048511

**Published:** 2012-10-31

**Authors:** Triinu Juurik, Heili Ilves, Riho Teras, Tanel Ilmjärv, Kairi Tavita, Kärt Ukkivi, Annika Teppo, Katren Mikkel, Maia Kivisaar

**Affiliations:** Department of Genetics, Institute of Molecular and Cell Biology, Tartu University and Estonian Biocentre, Tartu, Estonia; Louisiana State University and A & M College, United States of America

## Abstract

It is still an open question whether mutation rate can vary across the bacterial chromosome. In this study, the occurrence of mutations within the same mutational target sequences at different chromosomal locations of *Pseudomonas putida* was monitored. For that purpose we constructed two mutation detection systems, one for monitoring the occurrence of a broad spectrum of mutations and transposition of IS element IS*1411* inactivating LacI repressor, and another for detecting 1-bp deletions. Our results revealed that both the mutation frequency and the spectrum of mutations vary at different chromosomal positions. We observed higher mutation frequencies when the direction of transcription of the mutational target gene was opposite to the direction of replisome movement in the chromosome and *vice versa*, lower mutation frequency was accompanied with co-directional transcription and replication. Additionally, asymmetry of frameshift mutagenesis at homopolymeric and repetitive sequences during the leading and lagging-strand replication was found. The transposition frequency of IS*1411* was also affected by the chromosomal location of the target site, which implies that regional differences in chromosomal topology may influence transposition of this mobile element. The occurrence of mutations in the *P. putida* chromosome was investigated both in growing and in stationary-phase bacteria. We found that the appearance of certain mutational hot spots is strongly affected by the chromosomal location of the mutational target sequence especially in growing bacteria. Also, artificial increasing transcription of the mutational target gene elevated the frequency of mutations in growing bacteria.

## Introduction

Mutations provide a raw material for population diversity and adaptation; therefore the mutation rate has profound effects on population diversity and evolution dynamics. Mutagenesis is a complex multi-step process involving DNA target sequences and enzymes that play roles in DNA precursor metabolism, DNA replication, recombination and repair [1]. To elucidate molecular mechanisms of mutagenesis, it is necessary to develop systems that enable detection and analysis of mutations in living cells. Hence, over the years, a number of detection systems have been developed to study mutagenesis in bacteria [2]. Some of these mutation detection systems allow to monitor mutations in plasmids whereas others in bacterial chromosome.

Occurrence of mutations in plasmids and a chromosome may differ due to distinct replication mechanisms and higher copy number of plasmids in comparison to the chromosome. For instance, ColE1 plasmids do not replicate in the same manner as the *E. coli* chromosome [3]. Also, expression of plasmid conjugal functions may facilitate mutagenic processes, as it happens in the case of the occurrence of Lac^+^ revertants on F’ plasmid in *E. coli* FC40 strain [4], [5], [6]. On the other hand, when studying mutagenic processes in the bacterial chromosome, one should also consider various factors affecting DNA replication and thereby mutagenesis. A growing body of evidence have accumulated over the last years showing that replication inhibition by natural impediments such as DNA binding proteins, transcription units, unusual DNA structures and replication slow zones causes genomic instability [7]. Several other studies have addressed the effects of asymmetry of DNA replication on mutation rate [e. g., reviewed in [8]]. One DNA strand (the leading strand) is synthesized continuously, whereas the complementary strand (the lagging strand) is synthesized discontinuously in short Okazaki fragments. It has been suggested that different enzymology within the replication of two strands could provide a basis for different fidelity. For example, measurement of *lac* reversion frequencies for the two orientations in the chromosome of *E. coli* in the absence of DNA mismatch repair indicated that the lagging strand replication may be more accurate than leading strand replication [9], [10]. Yet, the results of subsequent studies have been contradictory. Under the conditions of constitutive expression of the SOS system the replication of the lagging strand appeared to be the major source of the mutations [11], whereas the following studies demonstrated lack of strand bias in UV-induced mutagenesis in *E. coli* [12].

The effect of chromosomal position on mutation rate has also been addressed in previous studies. Based on the results of comparison of synonymous substitution rates of a set of homologous genes in different bacterial species it was proposed that mutation rate depends on the distance of the target genes from the origin of replication in bacterial chromosome, being higher when genes are situated closer to the terminus [13], [14]. However, experimental studies have failed to detect such effect of the distance from the origin of replication on the mutation rates when *lacZ* alleles were inserted at four sites in the *Salmonella* genome [15]. At the same time, the reversion rates of the *lacZ* alleles inserted at an intermediate locus were significantly higher than those at loci nearer to and farther from the replication origin [15]. Recently, Martina et al. [16] demonstrated that frameshift mutation rate differs at distinct chromosomal positions but no correlation between the mutation rate and the distance of the mutation site to the origin of replication was found. In contrary to the above-mentioned studies, no significant mutational bias on the occurrence of base substitutions with regard to chromosome position or leading and lagging strands of replication were found in the two sequenced genomes of 5000 generations evolved *Salmonella typhimurium* under the conditions of reduced selection and in the absence of major DNA repair systems [17]. Also, the recent analysis of the genomes of 20 commensal and pathogenic *E. coli* strains suggests that the higher mutation rates estimated near the terminus could actually be associated with a reduced efficiency of selection due to lower rates of recombination at this region [18]. Thus, some controversy between the results of experimental approaches and the analyses of whole genome DNA sequences still exists and therefore the effect of the chromosome location on the rate of mutations needs further examination.

The growth phase of bacteria may also affect mechanisms of mutagenic processes. In nature, bacteria are very often confronted with variable and stressful environments. Under such conditions bacteria grow very slowly if at all [19], [20]. However, despite the reduced amount of DNA replication mutants arise that are able to take over bacterial populations. This process is known as adaptive mutagenesis, stress-induced mutagenesis or stationary-phase mutagenesis [21], [22]. It has been suggested that a variety of environmental stresses induce genomic change in bacteria, generating occasional fitter mutants and potentially accelerating the evolution of bacterial populations [21], [22], [23], [24], [25], [26]. Alternatively, the amplification model proposes that selection detects small improvements in growth due to a higher copy number of the selected gene; in that way mutation is made more likely because more copies of mutation target gene is added to each developing clone [27]. Despite the controversy in interpretations of the rate of mutations in stationary-phase populations the results of several studies have demonstrated that the spectrum of stationary-phase mutations is different from that occurring among the mutants of actively growing bacteria [28], [29], [30], indicating that distinct mechanisms are responsible for the appearance of mutations in actively growing and stationary-phase populations.

So far, *E. coli* has served as the primary model in virtually all fundamental aspects of microbiology including mutagenesis and evolution. However, recent advances in sequencing and annotation of more than a thousand of bacterial genomes have revealed that *E. coli* is rather exceptional considering its DNA polymerases and DNA repair enzymes [31], [32], [33], [34]. For example, *E. coli* is one of the rare organisms harboring DNA polymerase Pol V genes in its chromosome [32]. At the same time, pseudomonads, one of the largest groups of bacteria including both pathogenic and non-pathogenic species, possess some DNA polymerases and DNA repair enzymes which are widely represented in many bacteria except enterobacteria [35], thereby serving as a good model to study mutagenic processes in microorganisms distinct from *E. coli*. Unfortunately, a wide array of assay systems applicable for the detection of mutations in enterobacteria (e.g., those which are based on the activation of *lac* alleles) does not work in pseudomonads. Moreover, the test systems which have been available for the detection of mutations both in growing and stationary-phase populations of pseudomonads have enabled to monitor mutations on a plasmid [29], [36].

In the present study we have constructed novel chromosomal mutation detection systems for pseudomonads. These test systems can be inserted randomly into various chromosomal sites. Using these test systems in *P. putida* we have investigated: (i) the frequency of mutations at different chromosomal sites; (ii) the effect of orientation of the target gene on mutagenic processes in the bacterial chromosome; (iii) the effect of the growth phase of bacteria on mutagenic processes in the bacterial chromosome. Our results suggest that both the mutation rate and the spectrum of mutations vary at different chromosomal positions. Also, the chromosomal location of the mutational target has a larger effect on the occurrence of mutations in growing bacteria than in stationary-phase bacteria.

## Results

### Construction of Test Systems Detecting Mutations in the *P. putida* Chromosome

So far, only plasmidial test systems have been available to study the mechanisms of the occurrence of stationary-phase mutations under carbon starvation conditions of *P. putida* and other pseudomonads [29], [36]. These test systems have not been applicable for the detection of mutations in the bacterial chromosome because of too low mutation frequency. To overcome these limitations, we constructed two novel, more sensitive assay systems that allow studying mutational processes in *P. putida* chromosome both in growing and stationary-phase bacteria. Mutants able to grow on minimal medium containing phenol as only carbon and energy source (Phe^+^ mutants) can be isolated.

The phe-lacI test system detects different types of mutations either inactivating LacI repressor or abolishing its binding sites at the *lac* operator. In this test system the phenol degradation genes *pheBA* are placed downstream of the P*_tac_* promoter, which is negatively controlled by the LacI repressor. Details for the construction of the *lacI*-P_tac_-*pheBA* gene cluster (the phe-lacI test system) are shown in Materials and Methods and in Table S1. The second test system, pheA+C, was constructed to monitor a single type of Phe^+^ revertants at the fixed position of the gene *pheA*. This novel chromosomal test system is analogous to the plasmidial test system constructed by us earlier to measure reversion of a +1 frameshift mutation at a three C-nucleotides repeat flanking the inserted A nucleotide within the coding sequence of the *pheA* gene [36]. It is known that the frequency of frameshifts within nucleotides repeat is usually proportional to its length [37]. Therefore, to establish higher frequency of mutations than was achieved with the previously constructed plasmidial test system, an additional C nucleotide was introduced into a six C-nucleotides repeat at position 221 relative to the translation initiation codon of the *pheA* sequence. The frameshift mutation-containing *pheA* allele was transcribed from the constitutively expressed P*_gc_* promoter. Like the phe-lacI test system, it was inserted into the chromosome with mini-Tn*5* (see Materials and Methods and Tables S1 and S2 for the construction of this test system). Initial monitoring of the appearance of Phe^+^ revertants on phenol minimal plates containing 1×10^9^ tester cells of *P. putida* strains carrying the pheA+C test system at distinct sites in the chromosome revealed that this test system is very sensitive for the detection of chromosomal mutations. For instance, on day 6 after the plating of the tester cells thousands of Phe^+^ colonies had emerged onto phenol minimal plates. The occurrence of the 1-bp deletion at the expected site was confirmed by the sequencing of the *pheA* gene in 8 independently isolated Phe^+^ revertants.

### The Frequency of Mutations is Affected by the Chromosomal Location and Orientation of the Test System

**Figure 1:**
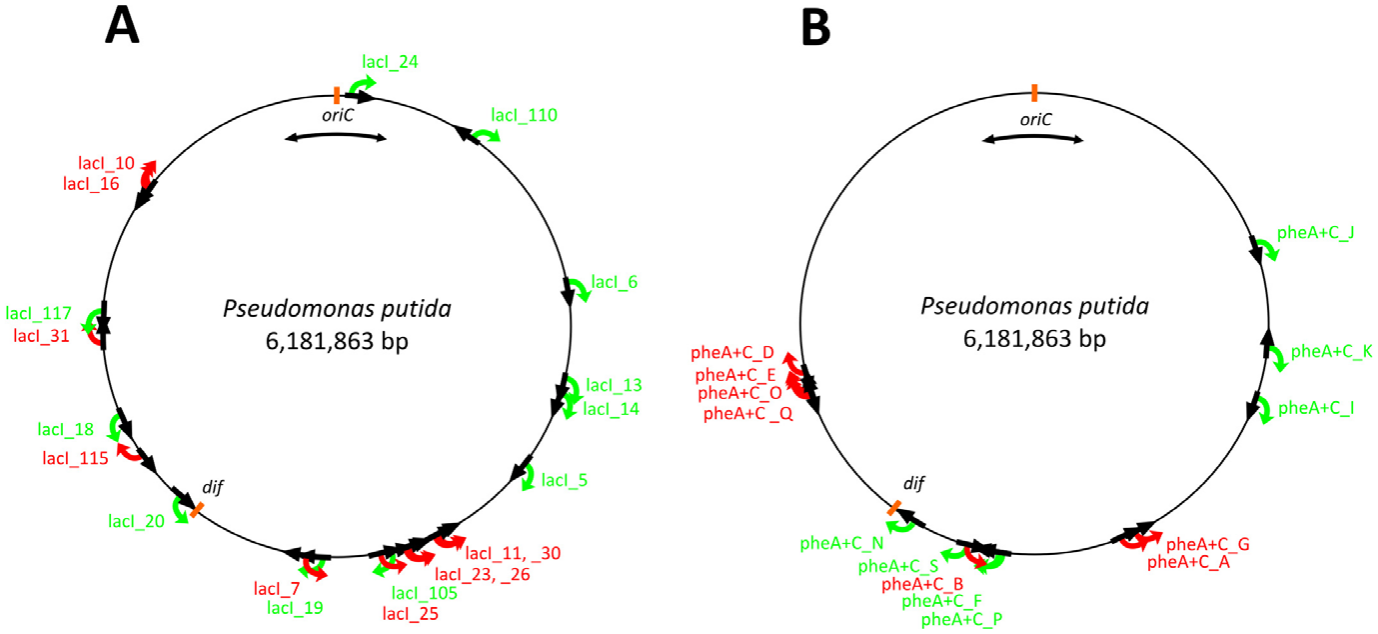
Chromosomal location of the randomly inserted test system in various *P. putida* strains. The locations of the phe-lacI test system detecting mutations which inactivate LacI repressor are shown in panel A (designated as lacI) and the locations of the pheA+C test system detecting only frameshift mutations are shown in panel B. The black arrows demonstrate the direction of transcription of the *P. putida* chromosomal genes containing the insertions of the test system. When the transcribed strand is the leading strand template for replication, the RNA polymerase and the replisome move in the same direction (co-directional orientation); when the transcribed strand is the lagging strand template, the RNA polymerase and the replisome converge (head-on orientations). The direction of transcription of the mutational target genes (the *lacI* gene in the phe-lacI test system and the *pheA* gene in the pheA+C test system) in different *P. putida* strains is indicated by green or red arrows. The red arrows designate head-on orientations of the transcription of the mutational target gene and the movement of the replisome in the chromosome and the green arrows point to co-directional transcription and replication. The replication of the chromosome starts at *oriC* region (indicated by two-directional arrow) and terminates at *dif* sites. Location of *P. putida dif* sequence is according to [87].

To explore the occurrence of mutations in different chromosomal locations of *P. putida*, we took 21 randomly selected chromosomal insertions of the phe-lacI test system and 14 random insertions of the pheA+C test system and monitored the accumulation of Phe^+^ mutants in these strains. The chromosomal location of the phe-lacI and pheA+C test systems in individual strains is shown in Fig. 1 and the exact insertion sites of the test system-carrying mini-Tn*5* are presented in Tables S3 and S4. We plated about 1×10^7^ tester cells together with 1×10^9^ scavenger cells onto selective plates containing phenol as an only carbon source and monitored the appearance of Phe^+^ mutant colonies in starving populations during 9 days.

In populations carrying the phe-lacI test system in most cases the first Phe^+^ colonies emerged onto selective plates on day 3 after the plating. Then, there was a 3-days period of rapid emergence of the mutants (Table 1). For days 8 and 9 the rate of the accumulation of Phe^+^ mutants significantly declined in all strains (the only exception was the strain phe-lacI_8 with the peak on day 8).

**Table 1:** The frequency of accumulation of Phe^+^ mutants in *P. putida* strains carrying the phe-lacI test system at different chromosomal locations^a^.

Strains^b^	HG^c^	Days							Total
		3	4	5	6	7	8	9	
**phe-lacI_30**	**(a)**	**17.12 (19.95)**	**49.00 (23.10)**	**35.84 (48.04)**	**56.28 (24.81)**	**17.04 (6.28)**	**8.41 (4.76)**	**12.67 (15.51)**	**196.35 (42.25)**
**phe-lacI_16**	**(a)**	**16.07 (9.11)**	**23.56 (16.90)**	**22.89 (15.62)**	**74.67 (48.58)**	**17.41 (11.51)**	**15.11 (8.22)**	**5.85 (4.25)**	**175.56 (87.76)**
**phe-lacI_25**	**(a)**	**4.49 (4.76)**	**103.29 (99.11)**	**11.12 (4.69)**	**34.80 (15.52)**	**9.99 (6.30)**	**3.61 (1.78)**	**2.50 (2.60)**	**169.8 (100.75)**
phe-lacI_117	(a)	2.75 (1.82)	36.93 (11.39)	43.13 (12.62)	50.97 (12.09)	22.89 (12.37)	4.27 (2.02)	2.44 (0.98)	163.48 (39.64)
**phe-lacI_11**	**(ab)**	**21.27 (47.43)**	**35.72 (26.00)**	**12.10 (10.81)**	**61.34 (43.82)**	**19.46 (12.55)**	**5.83 (2.47)**	**3.10 (4.25)**	**158.82 (94.14)**
**phe-lacI_115**	**(a)**	**0.45 (0.57)**	**63.31 (10.71)**	**17.78 (4.55)**	**30.24 (7.17)**	**17.55 (7.99)**	**5.32 (1.79)**	**1.81 (0.87)**	**136.47 (16.41)**
**phe-lacI_31**	**(ab)**	**10.91 (4.61)**	**30.25 (15.05)**	**13.79 (9.50)**	**49.71 (36.18)**	**8.98 (4.06)**	**5.79 (2.35)**	**5.91 (4.24)**	**135.23 (62.83)**
**phe-lacI_26**	**(ab)**	**14.92 (23.93)**	**43.02 (27.66)**	**13.83 (4.95)**	**36.41 (10.36)**	**11.03 (4.96)**	**2.37 (1.68)**	**6.69 (5.72)**	**128.27 (67.26)**
phe-lacI_12	(ab)	11.81 (11.26)	18.44 (13.36)	39.89 (33.47)	22.81 (9.43)	6.44 (6.24)	9.37 (6.77)	15.33 (9.35)	124.11 (53.01)
phe-lacI_105	(ab)	0.10 (0.23)	3.60 (3.67)	3.40 (3.62)	7.00 (6.50)	63.90 (10.73)	23.50 (4.39)	12.40 (2.64)	113.90 (18.11)
phe-lacI_110	(ab)	3.10 (1.52)	17.99 (5.10)	13.96 (5.59)	52.95 (12.06)	16.34 (6.11)	3.93 (1.67)	2.48 (1.22)	110.75 (20.45)
**phe-lacI_10**	**(ab)**	**10.41 (11.25)**	**19.46 (5.47)**	**10.76 (3.31)**	**53.42 (19.79)**	**7.68 (5.62)**	**4.07 (1.73)**	**4.56 (3.41)**	**110.35 (30.44)**
**phe-lacI_23**	**(ab)**	**5.2 (2.93)**	**30.08 (9.78)**	**12.17 (2.68)**	**31.81 (7.05)**	**14.41 (5.09)**	**3.38 (1.36)**	**4.59 (1.6)**	**101.88 (18.44)**
phe-lacI_14	(ab)	1.41 (1.25)	20.54 (10.65)	12.79 (5.39)	32.31 (21.29)	9.26 (4.92)	5.38 (4.37)	6.10 (2.91)	87.78 (37.81)
phe-lacI_6	(ab)	17.23 (22.02)	17.12 (10.72)	13.96 (7.33)	20.54 (7.69)	4.75 (2.98)	5.51 (5.10)	8.59 (3.90)	87.69 (38.41)
phe-lacI_8	(ab)	0.34 (0.78)	9.47 (9.11)	14.38 (12.49)	13.29 (12.05)	6.43 (5.25)	34.70 (35.18)	4.10 (3.27)	82.71 (35.19)
**phe-lacI_7**	**(ab)**	**0.74 (1.71)**	**23.65 (19.75)**	**12.40 (11.01)**	**29.62 (26.90)**	**8.78 (12.91)**	**2.48 (5.08)**	**3.33 (4.44)**	**81.01 (48.28)**
phe-lacI_13	(ab)	3.40 (4.56)	20.54 (9.24)	12.51 (7.60)	27.33 (16.47)	4.38 (2.80)	2.91 (1.81)	8.49 (3.82)	79.56 (26.08)
phe-lacI_24	(ab)	4.11 (1.87)	11.45 (4.56)	11.10 (5.83)	22.16 (16.85)	3.70 (3.31)	3.77 (1.56)	15.39 (16.07)	71.67 (21.86)
phe-lacI_1	(ab)	1.73 (2.85)	2.83 (2.23)	4.49 (2.28)	11.83 (4.76)	35.71 (10.04)	3.69 (2.03)	7.87 (4.07)	68.15 (13.76)
phe-lacI_5	(b)	5.23 (8.82)	9.13 (21.06)	12.27 (10.56)	14.69 (9.87)	5.12 (8.29)	3.43 (4.44)	7.34 (8.55)	57.21 (36.17)
phe-lacI_20	(b)	0.37 (0.38)	15.8 (5.60)	9.09 (3.00)	17.92 (4.4)	4.47 (1.00)	3.77 (1.92)	6.08 (2.48)	54.28 (8.73)
phe-lacI_19	(b)	0.24 (0.37)	17.13 (10.34)	6.40 (3.87)	21.21 (11.18)	3.76 (2.89)	1.49 (1.19)	2.08 (1.63)	52.29 (13.56)
phe-lacI_18	(b)	0	6.76 (2.22)	5.93 (1.74)	14.93 (3.02)	4.12 (1.44)	2 (1.06)	4.31 (2.12)	38.05 (5.91)

a Average number of Phe^+^ mutants per day and total number of mutants calculated per 1×10^7^ cells with 95% confidence intervals are shown. The results with at least 7 (7–28) independent populations of each strain are presented.

b Strains carrying the mutational target gene opposite to the direction of the movement of replisome in the chromosome are indicated in bold. Note that location of the test system in the chromosome of the strains phe-lacI_1, 8 and 12 has remained unknown.

c Homogeneity group.

The frequency of the accumulation of Phe^+^ mutants onto selective plates differs in the studied strains (Table 1). Based on ANOVA with *post-hoc* Tukey HSD test, the total numbers of Phe^+^ mutants accumulated for day 9 in the strains phe-lacI_30, phe-lacI_16, phe-lacI_25, phe-lacI_117 and phe-lacI_115 were separated to homogeneity group distinct from that for the strains phe-lacI_5, phe-lacI_18, phe-lacI_19 and phe-lacI_20 (*P*<0.05).

The phe-lacI test system enables detection of a broad spectrum of mutations which may have dissimilar effects on the growth rate of Phe^+^ colonies on phenol minimal plates. This complicates monitoring of dynamics of occurrence of mutations. In contrast, the pheA+C test system detects only a single type of mutations at the specific site, 1-bp deletions restoring the reading frame of the *pheA* gene. Thus, the usage of the pheA+C test system could allow better observation of changes in dynamics of occurrence of mutations in growing and carbon-starved *P. putida*. In general, it took approximately 4–6 days for first Phe^+^ colonies to appear onto selective plates when the *P. putida* strains carrying the pheA+C test system were examined for the accumulation of Phe^+^ mutants (Table 2). However, in the populations of the strain pheA+C_S, the first Phe^+^ mutants appeared on selective plates on day 11. For day 15, in average 5.7 mutants per 1×10^7^ viable cells was counted. Also, in average 12.5 mutants per 1×10^7^ cells emerged to selective plates during the days 11–15 in the populations of the strain pheA+C_P.

**Table 2:** The frequency of accumulation of Phe^+^ mutants in *P. putida* strains carrying the pheA+C test system at different chromosomal locations^a^.

Strains^b^	HG^c^	Days						Total
		4	5	6	7	8	9	
**pheA+C_B**	**(a)**	**0.34 (0.55)**	**0.9 (1.13)**	**4.85 (6.10)**	**36.25 (27.32)**	**55.21 (45.51)**	**6.95 (8.80)**	**104.5 (81.14)**
**pheA+C_O**	**(b)**	**0**	**0.24 (0.60)**	**20.50 (11.87)**	**6.96 (4.50)**	**0.24 (0.60)**	**0**	**27.93 (15.05)**
**pheA+C_G**	**(bc)**	**0**	**0**	**18.58 (8.83)**	**4.40 (2.96)**	**0.9 (1.13)**	**0**	**23.87 (10.25)**
**pheA+C_Q**	**(bc)**	**0**	**0**	**9.03 (7.68)**	**9.42 (2.38)**	**1.04 (0.922)**	**0**	**19.48 (5.59)**
**pheA+C_E**	**(bc)**	**0**	**0**	**6.25 (4.57)**	**8.24 (4.41)**	**0.70 (1.24)**	**0.51 (0.83)**	**15.71 (7.55)**
**pheA+C_A**	**(bc)**	**0**	**5.96 (2.93)**	**8.10 (3.85)**	**0.53 (0.62)**	**0.11 (0.27)**	**0**	**14.71 (5.2)**
**pheA+C_D**	**(bc)**	**0**	**0**	**7.01 (3.65)**	**3.66 (1.5)**	**0.29 (0.47)**	**0**	**10.96 (3.82)**
pheA+C_I	(bc)	0	2.91 (1.07)	7.71 (3.63)	0.21 (0.54)	0	0	10.83 (3.83)
pheA+C_N	(c)	0	0	0	0	8.07 (3.59)	1.71 (1.97)	9.77 (4.38)
pheA+C_F	(cd)	0	0	0	3.98 (2.49)	0.99 (0.90)	0.19 (0.48)	5.16 (3.38)
pheA+C_K	(d)	0	1.89 (1.59)	0.68 (1.17)	0	0	0	2.57 (1.97)
pheA+C_J	(d)	0	0	1.26 (0.94)	0.29 (0.48)	0	0.29 (0.48)	1.85 (1.30)
pheA+C_P	(d)	0	0	0.3 (0.77)	0	0	0	0.3 (0.77)
pheA+C_S	(d)	0	0	0	0	0	0	0

a Average numbers of Phe^+^ mutants per day and total number of mutants for day 9 calculated per 1×10^7^ cells with 95% confidence intervals are shown. The results with at least 6 independent populations of each strain are presented.

b Strains carrying the mutational target gene opposite to the direction of the movement of replisome in the chromosome are indicated in bold.

c Based on the comparison of the frequency of Phe^+^ mutations accumulated for day 9 statistically significantly different (*P*<0.05) homogeneity groups (a–d) appeared.

In general, the rate of the accumulation of Phe^+^ colonies declined approximately by one order of magnitude 4–5 days after the appearance of first colonies (Table 2). We observed the similar reduction in the emergence of Phe^+^ mutants in comparison to that at initial period also in this case when 10-times smaller amounts of the pheA+C tester cells were plated (data not shown). This indicated that the decline in the rate of accumulation of the 1-bp deletion mutants during the prolonged starvation was not caused by an inhibitory effect of the already existing Phe^+^ colonies on the growth of later appearing mutant colonies. Thus, our results demonstrated that the frameshift mutations occur at higher frequency in the *P. putida* chromosome when bacteria are still growing or have been only for a short time under conditions of carbon starvation. Later, during the prolonged starvation of bacteria the frequency of these mutations is remarkably reduced.

Similarly to the results obtained with the phe-lacI test system, the frequency of the occurrence of mutations detected by the pheA+C test system was influenced by the location of the test system in the chromosome (Table 2). Based on the comparison of the total number of the Phe^+^ mutants accumulated per 1×10^7^ cells for the day 9 it is possible to distinguish homogeneity groups with statistically significantly different mutant frequency. The strain pheA+C_B exhibited the highest mutant frequency (group a) and the strains pheA+C_K, pheA+C_J and pheA+C_P exhibited the lowest frequency of Phe^+^ mutatnts (group d). As already mentioned above, we detected the fast emergence of Phe^+^ mutants in the strains pheA+C_P and pheA+C_S at later incubation period (on days 11–15). However, the total number of the Phe^+^ mutants accumulated per 1×10^7^ for day 20 in the populations of the strains pheA+C_P and pheA+C_S remained statistically significantly lower than that in some other strains (e.g., in the strains pheA+C_B, O, and G) (data not shown).

To explain the differences in the rate of accumulation of Phe^+^ mutants in *P. putida* strains carrying the phe-lacI or pheA+C test system at distinct chromosomal sites, we examined the possibility that insertion of the test system into certain sites of the chromosome has altered the expression of genes which may influence mutation frequency in bacteria. However, based on the annotation of *P. putida* KT2440 genes at http://www.pseudomonas.com none of the genes which were targeted by the test system in the strains either exhibiting lower or higher Phe^+^ mutant frequency were known to be connected with processes which could affect mutation frequency (Tables S3 and S4). Additionally, to exclude the possibility that the differences observed in the Phe^+^ mutant frequency between individual strains could be a consequence of changes in overall mutation frequency, we compared the frequency of appearance Rif^r^ mutants in the strains that exhibited statistically significantly higher or lower Phe^+^ mutant frequency than the others. Among the strains carrying the phe-lacI test system, all strains belonging to the homogeneity groups a or b (Table 1) were examined. The strains phe-lacI_11 and phe-lacI_31 belonging to the homogeneity group ab were also included to this comparison. The average number of Rif^r^ mutants per 1×10^9^ cells was similar in majority of these strains (Table S5). According to the ANOVA with *post-hoc* Tukey HSD test, statistically significant differences appeared in the frequency of Rif^r^ mutants only in this case when the strain phe-lacI_117 was compared with the phe-lacI strains 5, 15, 20 and 30 (*P*<0.005). However, in contrary to the reduced frequency of Rif^r^ mutants, this strain expressed elevated frequency of Phe^+^ mutants (Table 1). The strains phe-lacI_105 and phe-lacI_110 also exhibited slightly reduced frequency of Rif^r^ mutants in comparison to the strain phe-lacI_16 (*P* = 0.047 and *P* = 0.039, respectively) but these differences could be insignificant since the *P*-values were close to α level (α = 0.05). The frequency of Rif^r^ mutants measured in the pheA+C test system-carrying strains (Table S6) did not differ statistically significantly or the *P*-value was close to α level (compare Rif^r^ mutant frequency in the strains pheA+C_G pheA+C_J, *P* = 0.037). Taken together these results suggested that the differences in the frequency of occurrence of Phe^+^ mutants in *P. putida* strains carrying the phe-lacI or pheA+C test system at distinct chromosomal sites are not caused by changes in overall mutation frequency. Rather, the location of the mutational target gene in the bacterial chromosome affects the frequency of occurrence of mutations in this gene.

To elucidate mechanisms which could affect the frequency of mutations at different chromosomal positions, we examined the effect of orientation of the target gene on mutagenic processes in the bacterial chromosome. Indeed, we found that the frequency of mutations was affected by the orientation of the mutational target gene in the chromosome. The rate of the accumulation of Phe^+^ mutants was higher when the direction of transcription of the mutational target gene (*lacI* in the phe-lacI test system or *pheA* in the pheA+C test system) was opposite to the direction of the synthesis of the leading strand in the bacterial chromosome and *vice versa*, the mutant frequency was lower when the direction of transcription of the target gene and synthesis of the leading strand were co-directional (Fig. 1 and Tables 1 and 2). At the same time, the frequency of Rif^r^ mutants was not dependent on the orientation of the mutational target gene of the test system in the chromosome (Tables S5 and S6). We divided the studied strains into two groups according to the direction of transcription of the mutational target gene and movement of replication fork. Group one contained the strains with co-directional transcription and replication and group two the strains with head-on orientations of transcription of the target gene and movement of replisome. By using the Mann-Whitney U test we found that the frequency of Rif^r^ mutants was similar in the two groups (*P* values were 0.194 and 0.081 for the phe-lacI and pheA+C strains, respectively). At the same time, the Phe^+^ mutant frequency differed statistically significantly between the two groups for both test systems (*P*-values were 0.003 and 0.002 for the phe-lacI and pheA+C strains, respectively). Thus, our results indicated that the direction of transcription of the mutational target gene in respect to the replisome movement influences the rate of mutations in the chromosome of *P. putida*.

### Artificial Increase of Transcription of the Mutational Target Gene in the *P. putida* Chromosome Elevates Occurrence of Mutations in Growing Bacteria

It has been suggested that the severity of replication fork arrest due to head-on transcription correlates with the level of transcription and may cause mutations especially within highly expressed genes [38], [39]. Our results support the idea that head-on orientations of RNA polymerase and the replisome elevates mutation frequency (Table 1 and 2). In order to evaluate the effect of the level of transcription on mutation frequency in the *P. putida* chromosome, we designed an assay where the transcriptional level of the mutational target gene can be artificially changed. We modified the pheA+C test system by placing the transcription of the *pheA* allele under the control of the LacI repressor and the IPTG-inducible P*_tac_* promoter (for details of the construction of the *lacI*-P*_tac_*-*pheA*+C cluster, see Materials and Methods and Table S1). Note that in the absence of IPTG, the *pheA* allele is transcribed in this cluster from the constitutively expressed P*_gc_* promoter. Similarly to the other test systems, the obtained Ptac-pheA+C test system was inserted into the chromosome of *P. putida* with mini-Tn*5*. We selected two random insertions of the test system with co-directional orientation of the transcription of the *pheA* allele and the movement of replisome (the strains Ptac-pheA+C_2 and Ptac-pheA+C_13) and two with head-on orientation (the strains Ptac-pheA+C_3 and Ptac-pheA+C_4) for the next studies. For exact location of the mini-Tn insertions, see Table S7.

To examine the effect of IPTG on the level of transcription of the *pheA* gene within the constructed *lacI*-P*_tac_*-*pheA*+C cluster, we performed Western blot analysis of the expression of the *pheA*-encoded phenol monooxygenase in cells of Phe^+^ revertant of the strain Ptac-pheA+C_2 either grown in the presence or absence of 0.5 mM IPTG. The results presented in Fig. S2 confirmed that the level of expression of the *pheA* gene is elevated when IPTG was added into the growth medium of bacteria. However, the IPTG effects were not detectable in cells of stationary-phase bacteria which were carbon-starved for 3 days (72 hours) on agar plates (Fig. S2). Thus, the constructed test system enabled to examine the effect of artificial increase of transcription of the mutational target gene on mutation frequency in growing cells but not in starving bacteria.

To specify conditions for monitoring the occurrence of Phe^+^ mutants in growing cultures of the tester strains, we examined how long it takes for Phe^+^ mutant colonies to appear onto phenol minimal plates. To mimic the growth conditions used for the selection of Phe^+^ mutants, approximately 100–200 cells of the already existing Phe^+^ mutants were plated onto phenol minimal agar together with 1×10^9^ scavenger cells not able to grow on phenol. The Phe^+^ colonies were only barely visible on day 7 and well-detectable on day 8 after the plating.

**Figure 2:**
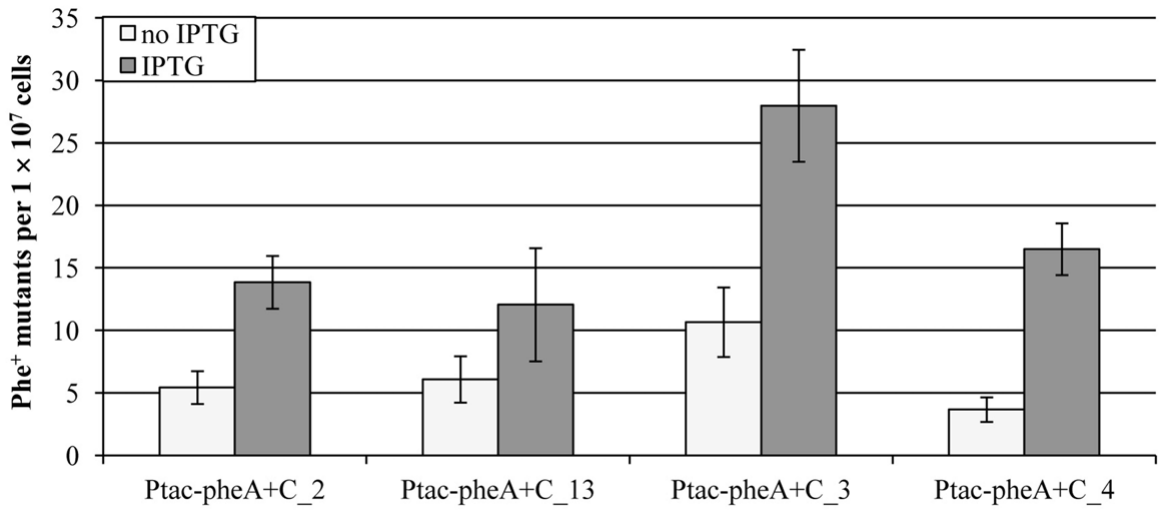
Effect of transcription of mutational target gene on mutation frequency. Influence of transcription was studied in growing bacteria. Average number of Phe^+^ mutants accumulated per 1×10^7^ viable cells is shown with 95%-confidence intervals. To compare the frequency of Phe^+^ mutants in growing *P. putida*, bacteria were grown in the presence or absence of 1 mM IPTG and the number of mutants emerged onto phenol minimal plates on day 8 were counted. In total, at least 30 independent cultures were examined in three parallel experiments for each strain.

To study whether the level of transcription of the mutational target gene could affect mutation frequency in *P. putida* growing cultures, we cultivated the tester strains in the presence or absence of IPTG before plating onto selective medium lacking IPTG. The number of Phe^+^ mutants appearing on selective plates on day 8 was compared. Despite the orientation of the mutational target gene *pheA* in the bacterial chromosome, we observed statistically significant increase (*post-hoc* Turkey HSD test) in the number of Phe^+^ mutants emerged on selective plates when bacteria were pre-grown in the presence of IPTG if compared to that when bacteria were grown in the absence of IPTG (*P*<0.001 for the strains Ptac-pheA+C_2, 3 and 4; *P* = 0.018 for the strain Ptac-pheA+C_13) (Fig. 2). Thus, the elevated level of transcription of the mutational target gene facilitated the frequency of mutations in growing bacteria.

### Spectrum of LacI-inactivating Mutations is Affected by the Chromosomal Location of the Target

**Figure 3:**
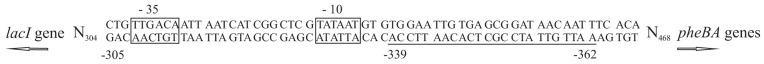
The location of the P*_tac_* promoter and the LacI operator sequence in the phe-lacI test system. −10 and −35 hexamers of the P*_tac_* promoter are boxed and the operator sequence is underlined. The nucleotide positions of the operator sequence are given from translational initiator codon GTG of the *lacI* gene.

Since the frequency of Phe^+^ mutations was altered at distinct chromosomal locations of the target gene, we investigated whether the frequency of occurrence of all mutations was similarly changed at particular loci or only certain types of mutations were affected. Therefore, we analyzed the spectrum of Phe^+^ mutations isolated in six different phe-lacI strains. Among the studied strains, phe-lacI_18, phe-lacI_105, phe-lacI_110 and phe-lacI_117 contained the mutational target gene *lacI* co-directionally and the strains phe-lacI_31 and phe-lacI_115 opposite to the direction of the replisome movement in the chromosome (Fig. 1A). The strains phe-lacI_115 and phe-lacI_117 expressed higher mutant frequency than several others, whereas the strain phe-lacI_18 exhibited the lowest rate of accumulation of Phe^+^ mutants among all phe-lacI test system-carrying strains characterized in this study (Table 1). We sequenced the *lacI* gene and the *lac* operator region (shown in Fig. 3) in Phe^+^ mutants which accumulated onto selective plates on days 3–7 (Table 3).

**Table 3:** Spectrum of Phe^+^ mutations in *P. putida* strains carrying the phe-lacI test system at various chromosomal positions.

Position^b^	Mutation	Number of occurrences in different phe-lacI strains^a^							
		105		110	115		117	18	31
−354	Del T		1 (0.709)	0		0	0	0	0
−353	Del 2 nt (TC)		1 (0.709)	0		0	1 (1.234)	0	0
−352	C → T		14 (9.927)	9 (8.381)		6 (6.746)	16 (19.74)	8 (1.951)	3 (2.622)
−352	C → G		2 (1.418)	0		0	0	0	0
−351	G → T		1 (0.709)	0		0	0	0	1 (0.874)
−351	G → A		13 (9.218)	26 (24.21)		27 (30.36)	16 (19.74)	38 (9.266)	38 (33.21)
−349	T → G		0	0		0	0	1 (0.244)	0
−349	T → C		0	0		2 (2.249)	0	0	0
−348	C → A		0	0		1 (1.124)	0	0	0
−347	A → G		0	0		1 (1.124)	0	0	0
−347	Ins A		0	1 (0.931)		0	3 (3.702)	0	0
−346	C → T		0	1 (0.931)		0	1 (1.234)	0	0
−345	Ins C		0	0		0	1 (1.234)	0	0
−3	Del 288 nt		1 (0.709)	0		0	0	0	0
13	A → G		0	0		0	0	1 (0.244)	0
22	Ins 22 nt		1 (0.709)	0		0	0	0	0
24	T → C		1 (0.709)	0		0	0	0	0
26	T → C		0	0		0	0	1 (0.244)	0
29	C → T		0	0		0	1 (1.234)	0	0
37	Del G		0	1 (0.931)		0	0	0	0
44	T → C		0	0		0	0	0	4 (3.495)
49	Del T		0	0		0	0	2 (0.488)	0
51	Ins A		0	0		0	0	2 (0.488)	0
52	Ins T		0	0		0	0	1 (0.244)	0
53	A → C		0	1 (0.931)		0	0	0	0
53	A → G		0	1 (0.931)		0	1 (1.234)	0	0
54	G → T		0	0		1 (1.124)	0	0	0
55	A → G		0	3 (2.794)		0	4 (4.935)	4 (0.975)	0
62	C → T		3 (2.127)	0		0	2 (2.468)	1 (0.244)	0
75	C → G		1 (0.709)	0		0	0	0	0
89	T → C		0	1 (0.931)		0	0	0	0
96	Ins 2 nt (CG)		0	0		0	2 (2.468)	0	0
100	Ins 10 nt		0	0		0	1 (1.234)	0	0
111	Ins 10 nt		1 (0.709)	0		0	0	3 (0.732)	0
131	G → C		1 (0.709)	0		0	0	0	0
140	A → G		0	1 (0.931)		0	0	0	1 (0.874)
141	C → G		1 (0.709)	0		0	0	0	1 (0.874)
146	C → T		1 (0.709)	0		0	0	1 (0.244)	0
149	Ins T		0	0		0	0	1 (0.244)	0
158	C → A		0	1 (0.931)		0	0	0	0
167	T → A		0	0		0	0	4 (0.975)	0
170	C → T		0	0		1 (1.124)	1 (1.234)	0	0
178	C → T		4 (2.836)	0		0	5 (6.169)	0	0
221	Del A		0	0		0	19 (23.44)	0	0
232	G → T		0	0		0	1 (1.234)	0	0
253	Del T		0	0		0	0	1 (0.244)	0
253	Del 14 nt		0	0		0	1 (1.234)	0	0
291	G → T		2 (1.418)	0		0	0	0	0
299	Del 149 nt		0	1 (0.931)		0	0	0	0
300	Del 10 nt		0	0		0	0	7 (1.707)	0
310	Del G		0	0		1 (1.124)	0	0	0
313	G → C		0	1 (0.931)		0	0	0	0
313	G → T		1 (0.709)	0		0	0	0	0
391	C → T		0	1 (0.931)		0	0	0	0
394	Del 66 nt		1 (0.709)	0		0	0	0	0
399	Ins C		0	0		0	1 (1.234)	0	0
403	Ins T		0	0		0	1 (1.234)	0	0
405	Ins T		1 (0.709)	0		0	0	0	0
409	Ins G		1 (0.709)	0		0	0	0	0
457	C → T		0	1 (0.931)		0	0	0	0
458	Ins C		0	0		1 (1.124)	0	0	0
460	Ins G		0	0		1 (1.124)	0	0	0
475	Ins T		0	0		0	1 (1.234)	0	0
476	Ins T		1 (0.709)	0		0	2 (2.468)	0	0
479	Ins T		1 (0.709)	0		0	0	0	0
490	Ins A		0	0		0	0	1 (0.244)	0
504	Ins G		0	0		0	0	0	3 (2.622)
541	C → T		0	0		0	0	2 (0.488)	0
552	Del G		1 (0.709)	0		0	0	0	0
592	Ins CTGG		3 (2.127)	3 (2.794)		58 (65.21)	1 (1.234)	6 (1.463)	65 (56.8)
592	Del CTGG		18 (12.76)	44 (40.98)		3 (3.373)	18 (22.21)	37 (9.022)	8 (6.991)
603	G → A		0	0		0	0	0	1 (0.874)
653	G → T		1 (0.709)	0		0	1 (1.234)	0	0
661	Ins C		1 (0.709)	0		0	1 (1.234)	0	0
669	Del G		0	0		0	1 (1.234)	1 (0.244)	0
682	C → T		0	0		0	0	1 (0.244)	0
705	Ins T		0	0		0	1 (1.234)	0	0
751	C → A		0	0		0	0	0	1 (0.874)
754	G → T		10 (7.091)	0		0	0	0	0
779	Ins 2 nt (CA)		0	0		1 (1.124)	0	0	0
780	C → A		1 (0.709)	0		0	0	0	0
858	A → C		0	0		0	1 (1.234)	0	0
916	C → T		0	2 (1.863)		0	0	0	0
983	C → T		0	0		0	1 (1.234)	0	0
IS1411			17 (12.05)	12 (11.18)		8 (8.995)	16 (19.74)	4 (0.975)	2 (1.748)
unknown			3 (2.127)	1 (0.931)		3 (3.373)	5 (6.169)	2 (0.488)	2 (1.748)
Overall mutation frequency			78	104.3		129.3	156.7	31.7	113.6
Total number analyzed			110	112		115	127	130	130

a Frequency of mutation per site calculated per total number of Phe^+^ mutants accumulated per 1×10^7^cells for day 7 is shown in parentheses.

b Positions of nucleotides are given in respect to the *lacI* coding sequence so that the first translated codon GTG is at position 1–3. Mutations at positions −345 to −354 alter the LacI operator sequence.

We detected various base substitutions and single-nucleotide insertions in the *lac* operator region, and base substitutions, insertions and deletions in the *lacI* repressor gene. CTGG insertions and deletions at position 592 of the *lacI* coding sequence and C-to-T or G-to-A transitions in the *lacI* operator region at the positions −352 and −351 relative to the translation initiation codon of the *lacI* gene in the phe-lacI test system occurred the most frequently. Additionally, we detected insertions of IS element IS*1411*. IS*1411* locates just downstream of the *pheBA* genes in the phe-lacI test system. We have previously shown that insertion of this element in proper orientation upstream of the promoterless *pheBA* genes activates transcription of these genes due to the presence of outward-directed promoters at the left end of IS*1411* [40]. In the current study we detected insertions of IS*1411* into the *lacI* gene resulting in inactivation of the LacI repressor.

Statistical analysis of the data using the Monte Carlo method devised by Adams and Skopek [41] established that the spectra of mutations identified in the six phe-lacI strains were different from each other (for *P* values, see the results of analysis of total spectrum in Table S8). The most significant differences appeared between the occurrence of CTGG insertions and deletions (indels) at the position 592 of the *lacI* coding sequence (see Ins CTGG and Del CTGG in Table 3 and Table S9). These differences were connected with the orientation of the *lacI* gene in the chromosome. CTGG deletions occurred at this site preferentially in the strains phe-lacI_105, phe-lacI_110, phe-lacI_117 and phe-lacI_18 which carry the mutational target gene *lacI* in the same direction as the movement of replication fork. CTGG insertions prevailed in the strains phe-lacI_115 and phe-lacI_31 which carry the mutational target gene in the opposite direction. Another mutational hot spot, the G-to-A transitions, was identified in the *lac* operator sequence at the position −351. The appearance of this mutation also varied depending on the chromosomal location of the test system. The −351 G-to-A transitions occurred in 30% cases among all Phe^+^ mutations identified in the strains phe-lacI18 and phe-lacI31 but in 12–13% cases in the strains phe-lacI_105 and phe-lacI_117. These differences were statistically significant (*P* = 0.001; Table S9). The proportion of the C-to-T transitions at the position −352 also significantly varied (e.g., compare phe-lacI_31 *versus* phe-lacI_105 and phe-lacI_117, *P* = 0.002; Table S9). Additionally, certain mutational hotspots appeared merely in one of the studied strains (Table 3). For example, the A nucleotide deletions at the position 221 was specific to the strain phe-lacI_117, the G-to-T transversions at the position 754 were detected only in the strain phe-lacI_105, and 10-nt deletions at the position 300 were characteristic to the strain phe-lacI_18. These differences were also statistically significant (*P*<0.001). Thus, our results demonstrated that not only the overall frequency of mutations but also the spectrum of mutations is affected by the chromosomal location of the target sequence.

The fact that the CTGG deletions prevailed in the strains which carried the mutational target gene *lacI* in the same direction as the movement of replication fork and the CTGG insertions appeared preferentially in the strains which carried the mutational target gene in the opposite direction was intriguing. To further confirm, that the orientation of the mutational target gene in the bacterial chromosome could affect the occurrence deletions and insertions, we extended our analysis of the generation of CTGG indels for additional six strains. Three strains used in this experiment carried the mutational target gene opposite to the direction of the movement of replisome and expressed the elevated frequency of Phe^+^ mutants (the strains phe-lacI_16, phe-lacI_25 and phe-lacI_30; see Table 1). The other three strains with co-directional transcription of the target gene and movement of replisome expressing the reduced frequency of Phe^+^ mutants (the strains phe-lacI_5, phe-lacI_19 and phe-lacI_20; Table 1) were also examined for the occurrence of CTGG indels. We found that similarly to the results obtained from our previous experiments (Table 3), the CTGG insertions prevailed in the strains carrying head-on orientation of the mutational target and the movement of the replisome (Table 4). On the other hand, the CTGG deletions prevailed in the strains phe-lacI_19 and phe-lacI_20 carrying the mutational target gene *lacI* in the same direction as the movement of replication fork (Table 4). Thus, the results obtained from the extended analysis of the occurrence of CTGG indels confirmed orientation-dependent effects on the generation of deletions or insertions in our assay.

**Table 4:** Effect of the orientation of mutational target gene in the chromosome on occurrence of CTGG indels in *P. putida* strains carrying the phe-lacI test systems at various chromosomal positions.

Position	Mutation^a^	phe-lacI strains^b^					
		5	16	19	20	25	30
592	Ins CTGG	32	**35**	9	10	**39**	**35**
592	Del CTGG	6	**2**	26	15	**3**	**5**
Unknown		3	**5**	11	17	**4**	**6**
Total number analysed		41	**42**	46	42	**46**	**46**

a Phe^+^ mutants accumulated on selective plates on days 3 and 4 were analysed for the occurrence of CTGG indels.

b Strains carrying the mutational target gene opposite to the direction of the movement of replisome in the chromosome are indicated in bold.

In addition to the differences in the occurrence of point mutations and indels, the frequency of transposition of IS*1411* was affected by the chromosomal location of the target site (Table 3). We detected the insertions of IS*1411* into the *lacI* gene only 4 and 2 times in the strains phe-lacI_18 and phe-lacI_31, respectively, but 8–17 times in the other 4 strains. Statistically significant differences (*P*<0.002) appeared when the frequency of transposition in the strain phe-lacI_31 was compared with that in the phe-lacI strains 105, 110, 115 and 117, and in the strain phe-lacI_18 with that in the phe-lacI strains 105, 110 and 117 (Table S6).

### Different Types of Phe^+^ Mutations Prevailed in Growing and Stationary-phase Bacteria

As already mentioned above, the DNA sequencing of the Phe^+^ mutants isolated in *P. putida* strains carrying the phe-lacI test system enabled us to identify a wide array of mutations which either inactivated the LacI repressor or abolished its binding site at the operator sequence (Table 3). Since Phe^+^ mutants continued to accumulate onto selective plates during prolonged incubation of bacteria, we supposed that in addition to Phe^+^ mutations that occurred in growing cells, before the plating, several others occurred during the starvation of bacteria on phenol minimal plates. Additionally, time-dependent appearance of the mutants onto selective plates could be caused by dissimilar effects of individual mutations on the level of transcription of the *pheA* gene: Phe^+^ mutants with complete inactivation of the LacI repressor could grow faster on phenol minimal plates than those with partial suppression of its functions. It was also possible that the occurrence of certain mutations is affected by physiological conditions of bacteria, i.e., some mutations arise preferentially in growing bacteria whereas some others in stationary-phase. Therefore, in order to investigate whether the growth phase of bacteria could influence the occurrence of mutations in *P. putida* chromosome, we analyzed the spectra of mutations of Phe^+^ mutants that emerged on the selective plates at different periods (on days 3–4, 5, and 6–7).

The spectrum of Phe^+^ mutations after different time periods is shown in Table 5 and Table S10. The most remarkable changes appeared in the occurrence of the CTGG indels at the position 592 of the *lacI* sequence and the −351 G-to-A transitions in the *lac* operator sequence The CTGG indels prevailed in all strains when the mutants emerged on days 3–4 were examined (Table 5). In contrary to that, the −351 G-to-A transitions were the most frequent mutations later, on days 6–7 (Table 5). It was possible that the mutations in the LacI repressor operator sequence (e.g., −351 G-to-A transitions) only partially abolished the binding of LacI to the operator. Such mutants could grow slower on phenol minimal plates than those with full inactivation of the *lacI* gene due to the CTGG indels which changed the reading frame of the *lacI* gene. In order to examine the effects of the CTGG indels and the −351 G-to-A transitions on the growth rate of the Phe^+^ mutants, we performed a reconstruction experiment. About 100–200 cells of the individual Phe^+^ mutants either containing the CTGG indels or the −351 G-to-A transitions were mixed with 1×10^9^ *P. putida* scavenger cells that were not able to grow on phenol minimal medium and the mixtures were plated onto phenol minimal plates. The appearance of single Phe^+^ colonies of these mutants, either derived from the strain phe-lacI_18 or phe-lacI_31, was monitored. The Phe^+^ mutant colonies which contained the CTGG indels appeared onto the phenol-containing minimal plates on day 3 after plating, whereas those which contained the −351 G-to-A transitions in the *lac* operator emerged one day later, on day 4. These results indicated that the appearance of different mutational hot spots among the mutations derived from earlier and later periods of incubation of bacteria on selective plates might be caused by dissimilar effects of these mutations for the growth of Phe^+^ colonies on phenol minimal plates.

**Table 5:** Time-dependent appearance of mutational hot spots in *P. putida* strains carrying the phe-lacI test system.

Days	Mutation position	pheA-lacI strains						
		105	110	115	117	18	31	
3–4	−352 C→T	0	0	0	0	3	0	
	−351 G→A	0	0	0	0	0	0	
	221 del A	0	0	0	19	0	0	
	592 ins CTGG	1	0	34	1	3	42	
	592 del CTGG	12	38	2	12	30	4	
	754 G→T	8	0	0	0	0	0	
	Ins IS*1411*	0	3	5	3	0	0	
	Total number	26	50	44	50	50	50	
5	−352 C→T	0	9	4	11	5	2	
	−351 G→A	1	0	0	1	0	0	
	221 del A	0	0	0	0	0	0	
	592 ins CTGG	0	1	15	0	2	15	
	592 del CTGG	4	4	0	3	4	4	
	754 G→T	2	0	0	0	0	0	
	Ins IS*1411*	8	5	1	2	4	2	
	Total number	32	25	24	27	30	30	
6–7	−352 C→T	14	0	2	5	0	1	
	−351 G→A	12	26	27	15	38	38	
	221 del A	0	0	0	0	0	0	
	592 ins CTGG	2	2	9	0	1	8	
	592 del CTGG	2	2	1	3	3	0	
	754 G→T	0	0	0	0	0	0	
	Ins IS*1411*	9	4	2	11	0	0	
	Total number	52	37	47	50	50	50	

Although the growth rate of the Phe^+^ colonies on the phenol minimal plates varied due to dissimilar effects of individual mutations on the transcription of the *pheA* gene from the P*_tac_* promoter, this was not the only reason that the mutational spectra derived from distinct time periods differed from each other. Whereas in the reconstruction experiments the colonies of the Phe^+^ mutants with the −351 G-to-A transitions at the *lac* operator sequence became visible on selective plates already on day 4 after the plating, these transitions prevailed in the late-appearing Phe^+^ mutants that emerged onto the selective plates later, on days 6–7 (Table 5). This implied that the G-to-A transitions could occur preferentially in populations of starving bacteria, after the plating of tester cells onto phenol minimal plates. In order to further explore whether the occurrence of certain types of mutations could be dependent on physiology of bacteria, we performed statistical analysis of the mutational spectra derived from different time periods and compared these spectra in each strain separately by using the Monte Carlo method. Because the *lac* operator mutations and *lacI*-inactivating mutations had dissimilar effects on the growth of Phe^+^ mutants on selective plates, we omitted the *lac* operator mutations from this analysis and focused only to *lacI* mutations. The Monte Carlo test revealed statistically significant differences in all strains (with the exception of the strain phe-lacI_115) when the spectrum derived from days 3–4 was compared with the spectrum of mutations from day 5 (Table S11). The differences appeared also in this case when the spectrum of mutations from the days 3–4 was compared with that from the days 6–7 in the strains phe-lacI_105 and phe-lacI_117. At the same time, except for the strain phe-lacI_105, no differences appeared when the mutational spectrum from the day 5 was compared with the spectrum from the days 6–7 (Table S11).

Importantly, statistically significant differences appeared also in this case when the spectra of mutations in different strains from the earlier time periods were compared with each other. However, differences were smaller or disappeared when the later periods were compared (Table S8 and S12). Thus, based on this analysis we suggest that more variability in the occurrence of mutations in the *P. putida* chromosome could appear at the stage of active growth of bacteria or when bacteria have spent only short period under the carbon starvation conditions.

The effect of the growth phase of bacteria on the occurrence of certain mutations can be illustrated also by two another DNA sequence alterations which inactivated the *lacI* gene. The A-nucleotide deletion at the position 221, which occurred specifically only in the strain phe-lacI_117, was detected in Phe^+^ colonies that emerged onto selective plates on days 3 and 4 but not later (Table 5 and Table S10). Also, the G-to-T transversions at the position 754, specific to the strain phe-lacI_105, occurred also as the hot spot in the early-arising mutants and were not detected when the later period (days 6–7) was investigated (Table 5 and Table S10). The frequent appearance of these two mutations in the early-arisen Phe^+^ colonies and their absence in the spectrum of mutations at the later periods indicated that these point mutations could occur preferentially in growing bacteria.

Taken together, the results of the analysis of the spectra of mutations from different time periods allowed us to draw two conclusions. First, some mutations in the *P. putida* chromosome occur preferentially in the growing cells and are rare in stationary-phase, whereas certain other mutations (e.g., −351 G-to-A transitions at the *lac* operator) prevail in stationary-phase bacteria. Secondly, the chromosomal location of the mutational target to a larger extent influences the occurrence of mutations in growing cells than in stationary-phase cells.

## Discussion

It has been known for many years that the mutation rate can vary dramatically between nucleotide sites [42]. Recent analysis of large genomic data sets of eukaryotic genomes suggests that the mutation rate can vary over many different scales, from the adjacent sites to whole chromosomes [43]. Compared to the complex organization of eukaryotic chromosomes, bacterial chromosomes are smaller and structurally simpler. It is still an open question whether the mutation rate can vary across the bacterial chromosome. Earlier studies have suggested that the genes farther from the replication origin have higher mutation rates than those nearest to it [13], [14]. Examination of the reversion rates of *lacZ* alleles inserted at four positions in the *Salmonella enterica* chromosome [15], however, did not support these findings. Nevertheless, significantly higher mutation frequency at intermediate locus than those inserted closer to replication origin or terminus appeared [15]. In contrast to the above-mentioned studies, recently published analysis of whole genome sequences suggests that there is no significant mutational bias with regard to chromosome position [17], [18].

With the advent of next-generation sequencing, powerful measurements of mutation rates are now possible using whole-genome sequences of isolates either sampled from evolution experiments or derived from natural isolates of the same or related species. However, such estimates meet difficulties in distinguishing between selection and neutral processes. Our experimental approach has enabled to monitor the occurrence of mutations within the same mutational target sequence (*lacI* or *pheA*) at many different chromosomal locations. In this case the effect of selection on the fixation of mutations is the same irrespective of the chromosomal location of the target sequence. Based on the results of the comparison of the frequency of occurrence of Phe^+^ mutations in the *P. putida* chromosome either carrying the phe-lacI or pheA+C test system at 21 and 14 positions, respectively (Table 1, Table 2) we suggest that the occurrence of mutations varies in different chromosomal loci. Comparison of the spectra of LacI inactivating mutations in six chromosomal positions of the phe-lacI test system revealed that these spectra were statistically significantly different from each other (Table 3, Table S7). These results altogether demonstrated that the occurrence of mutations is affected by the chromosomal location of the mutational target sequence. Several possible mechanisms may operate that can explain the differences observed in the current work.

### Effect of Head-on Transcription and Replication on Mutation Frequency

We did not notice any correlation between frequencies of mutations and distance of the studied sites from the origin of replication. Our results are in agreement with the recently published results of the comparison of rate of frameshift mutations (the assay detects loss of A•T from (A•T)_8_ repeat in chloramphenicol acetyl transferase gene) at different positions in the *E. coli* chromosome [16]. This work also demonstrated that mutation rate varies at different positions in the genome. However, in contrast to the assay used in [16] we observed higher mutation frequency when the direction of transcription of the mutational target gene was opposite to the direction of movement of the replisome in the chromosome and *vice versa*, lower Phe^+^ mutation frequency was accompanied with co-directional transcription and replication. This connection appeared either by testing the occurrence of the broad spectrum of mutations in *P. putida* strains carrying the phe-lacI test system in the chromosome or by monitoring one particular frameshift mutation in the range of pheA+C test system-carrying strains (Fig. 1, and Tables 1 and 2).

Several studies have indicated that co-directional transcription complexes do not impede replisome progression, whereas head-on collisions can result in replication fork arrest and may therefore induce DNA recombination and repair [44], [45], [46]. It has been suggested that the severity of replication fork arrest due to head-on transcription correlates with the level of expression and may cause mutations especially within highly expressed genes [38], [39]. Nevertheless, increased mutagenesis has been associated with head-on collisions also within genes that are transcribed at lower levels. For example, a recent *in vivo* study in *B. subtilis* demonstrated an increase in the *rpoB* mutation rate when the genomic region encoding the *rpoB* gene was inverted so that it was transcribed head-on to the replication [39]. Our observations on mutagenic processes taking place in *P. putida* chromosome support the idea that the head-on collisions between transcription and replication could elevate mutation frequency (Table 1 and 2). At the same time, it should be noted that the frequency of the accumulation of Phe^+^ mutants in the strain phe-lacI117 was also elevated if compared to the several other strains, although in this case the mutational target gene (*lacI* gene) was transcribed co-directional to the replication. Also, the orientation of the mutational target gene in the chromosome had no statistically significant effect in the emergence of Phe^+^ mutants in some other tester strains. These results imply that in addition to the effects caused by the co-directional or head-on orientations of RNA polymerase and the replisome, the rate of mutation in the distinct chromosomal sites might be affected by several other factors.

### Asymmetry in Mutagenesis during Leading and Lagging Strand Replication

The differences between the leading and lagging-strand replication have suggested that the production of mutations is not equal in the two strands. Based on the analysis of mutations in *lacZ* gene in two orientations in the *E. coli* chromosome, the lagging strand has been proposed to be less mutagenic [9]. Additionally, the base composition of DNA strands may affect the occurrence of mutations.

#### (i) Frameshift mutations at homopolymeric runs

Frameshift mutations can occur as a result of template-primer slippage at homopolymeric runs or repetitive sequences [47], [48]. *In vitro* experiments with eukaryotic DNA polymerases have shown that 1-bp frameshifts occur more frequently in runs of template pyrimidines (at runs of T or C) than in runs of purines (at runs of A or G) [37]. Additionally, asymmetry of frameshift mutagenesis during the leading and lagging-strand replication has been demonstrated in comparison of reversion of various *lacZ* alleles in two orientations in the *E. coli* chromosome showing that the frequency of occurrence of 1-bp frameshift at (C·G)_6_ run within the *lacZ* allele was higher when the template of the leading strand contained the run of pyrimidines [10]. This model could also be applied to explain the differences in mutation frequency in *P. putida* strains carrying the pheA+C test system at various chromosomal sites. The pheA+C test system measures the occurrence of 1-bp deletions at the (C·G)_7_ run starting in the *pheA* sequence at the position 221 relative to the translational initiator codon of this gene. The coding strand of the mutated *pheA* gene contains seven C nucleotides at this site. The effect of the orientation of the mutational target gene appeared most remarkably in the comparison of the frequency of accumulation of Phe^+^ mutations in the strains pheA+C_B and pheA+C_S. Specifically, these two strains contain the insertions of the test system into the same gene, *tnpS* (PP_2981), which encodes for cointegrase of the transposon Tn*4652*, but in different orientations (Fig. 1B). The insertion sites are very close to each other, separated only by 108 nucleotides. In the strain pheA+C_B the transcription of the mutational target gene *pheA* is opposed to the direction of the replication of the leading strand and the frequency of Phe^+^ mutations was significantly higher than that in the strain pheA+C_S in which the transcription of the *pheA* gene was co-oriented with the replication of the leading strand in the chromosome. If the *pheA* gene is transcribed in the same direction as the replication fork movement (as it happens in the strain pheA+C_S, and also in the strains pheA+C_K, pheA+C_J and pheA+C_P expressing reduced Phe^+^ mutation frequency in comparison with a number of others), the template for the leading strand synthesis contains the G-nucleotide run, while in the opposite direction (as it happens in the strain pheA+C_B) the C-nucleotide run serves as the template. Thus, similarly to the above-mentioned studies [10], [37] the higher frequency of the occurrence of Phe^+^ revertants in the strain pheA+C_B than in the strain pheA+C_S and also in some other strains could be explained by preferred occurrence of 1-bp deletions when the template of the leading strand contains pyrimidines.

#### (ii) Frameshift mutations at repetitive sequences

Among the mutations which inactivated the *lacI* repressor gene in our study, the largest fraction of mutations consisted of the deletions or insertions of the 4-nucleotide sequence CTGG which is tandemly repeated three times at the positions 592 to 604 in the *lacI* gene. The hotspot mutation at this site of the *lacI* gene has been demonstrated previously in *E. coli* [49], [50], [51]. Note that the numbering of nucleotide positions at the *lacI* sequence in the current work differs by 29 nucleotides from that used in the previous studies in *E. coli*: in the current study the first translated codon GTG is at position 1–3 instead of position 29–31. Interestingly, among the 12 phe-lacI strains analyzed by us, the CTGG insertions prevailed in the strains containing the CTGG template in the leading strand whereas the CTGG deletions occurred predominantly in the strains containing the CTGG template in the lagging strand (Table 3 and 4). The instability of tandem repeats has been attributed to DNA polymerase slippage at misaligned intermediates in which an extrahelical loop compromised of one or more repeat units is stabilized by the surrounding correct base-pairs [48], [52]. However, slippage potentiated by the CTGG repeated sequence in the *lacI* gene was supposed to be not the only misalignment that predicts the hotspot event [50].

It has been suggested that replication-dependent deletions between direct repeats occur preferentially in the lagging strand due to an unequal probability to form hairpin structures [53]. Also, the results of the another study have demonstrated that both expansions and deletions of CTG repeats occur in *E. coli* in an orientation-dependent manner [54]. In that study the deletions occurred more frequently when the CTG template was in the lagging strand whereas expansions were more prominent when the CTGs were in the leading strand template. Thus, we suggest that analogously to the mechanisms proposed for the gain and loss of CTG repeats [54] the orientation-dependent effects observed in the present study could be explained by the preferred formation of the deletion intermediates when the CTGG repeat in the *lacI* sequence is in the lagging strand template.

### Effect of Level of Transcription on Mutagenesis

There are several studies demonstrating that spontaneous mutation rate is proportional to the transcriptional level both in eukaryotic cells [55], [56] and in bacteria [57], [58], [59], [60], [61]. Thus, it is possible that in addition to the effects of the orientation, the effects of the level of transcription of the mutational target gene influenced the frequency of Phe^+^ mutations in our studies. For example, the strains pheA+C_B and pheA+C_S differed significantly from each other not only by the frequency of the occurrence of Phe^+^ revertants but also by the level of the expression of the *pheA* gene. The Phe^+^ mutants which accumulated in the strain pheA+C_S grew slower than those emerged in the strain pheA+C_B due to the lower cellular amount of the phenol monooxygenase PheA (Figure S1). The reason for the level of transcription of the *pheA* gene being reduced in the strain pheA+C_S is unclear. Although the direction of the transcription of the *pheA* gene opposed the direction of transcription initiated from the *tnpS* gene promoter in this strain, it is unlikely that transcription proceeding from the *tnpS* promoter could suppress transcription of the *pheA* gene. The pheA+C test system-carrying mini-transposon contains several other genes (e.g., those associated with tellurite resistance) in its other end, thereby separating the *pheA* gene from the *tnpS* promoter by a nearly 4-kb-long DNA segment.

In order to examine the possibility that the level of transcription of the mutational target gene could affect the frequency of mutations, the transcription of the mutational target gene in the pheA+C test system was placed under the control of IPTG-inducible P*_tac_* promoter. The increased level of transcription of the mutational target gene had statistically significant effect on the frequency of occurrence of frameshift mutations in growing bacteria (Fig. 2). Thus, we suggest that in addition to the DNA strand bias (e.g., higher frequency of mutations when the template for the lagging strand synthesis contains the G-nucleotide run) and transcription and replication collisions, changes at the level of transcription of the mutational target gene may affect mutagenic processes at least in growing cells of *P. putida*.

### Effect of Growth Phase of Bacteria on Mutagenic Processes in the Chromosome

We have monitored the occurrence of Phe^+^ mutations in the *P. putida* chromosome both in growing and in stationary-phase bacteria. Study of the dynamics of accumulation of Phe^+^ mutants revealed that after the initial fast period the emergence of Phe^+^ colonies onto selective plates decreased (Table 1 and Table 2). The decline in the accumulation of Phe^+^ mutants was more clearly visible with the pheA+C test system than that with the phe-lacI test system. The pheA+C test system scores only 1-bp deletions at the fixed position in the mutated *pheA* allele resulting in similar growth rate of Phe^+^ revertants on phenol minimal plates. Thus, the time-dependent emergence of Phe^+^ colonies on selective plates in *P. putida* carrying the pheA+C test system could reflect dynamics of the occurrence of mutations in the bacterial chromosome in growing and stationary-phase populations of *P. putida*. We suggest that the decline in the number of later-appearing Phe^+^ mutants might be caused by reduced replication of the chromosome under conditions of carbon starvation of bacteria.

The results of the current study differ remarkably from that observed by us previously with the plasmidial test systems when mutations accumulated onto selective plates at constant rate or even increased in starving populations of *P. putida* [29], [36], [62]. In these studies we have excluded the possibility that the copy number of the plasmid could be increased in starving bacteria, thereby facilitating occurrence of stationary-phase mutations. Thus, the differences in the dynamics of occurrence of mutations in the chromosome and in plasmid during prolonged incubation of *P. putida* on selective plates are not clear yet.

The usage of the phe-lacI test system enabled the simultaneous detection of a broad spectrum of mutations (Table 3, Table 5). The CTGG indels at the position 592 in the *lacI* gene were the most frequently detected mutations when the earlier-arising Phe^+^ colonies were investigated, whereas the −351 G-to-A transitions in the *lac* operator sequence prevailed among the late-arising Phe^+^ mutants (Table 5, Table S10). The Phe^+^ mutants carrying the −351 G-to-A transitions in the *lac* operator appeared in the reconstruction experiment onto phenol minimal plates one day later than those containing the CTGG indels, which demonstrates that the *lac* operator mutants grow slower. Nevertheless, since the emergence of G-to-A transitions on selective plates in the mutagenesis assay was delayed by about 3 days in comparison with that of mutants that arose due to the CTGG indels, we suggest that the −351 G-to-A transitions occur preferentially in stationary-phase cells. The appearance of certain mutational hot spots especially in stationary-phase cells of *P. putida* has been observed also in our earlier studies when we employed plasmidial test systems for the detection of mutations [29], [62]. A difference in the spectrum of mutations between stationary-phase and actively growing bacteria has been demonstrated also in *E. coli* using the FC40 system that detects reversion of the *lac* allele on F plasmid [28], [29], [30]. The results of the current work indicate that the occurrence of certain types of mutations preferentially in stationary-phase cells might be more general, encompassing mutagenic processes taking place also in the chromosome of *P. putida*.

Interestingly, our results imply that the occurrence of certain mutational hot spots especially in growing bacteria is affected by the chromosomal location of the mutational target sequence (Table 3 and 5 and Table S10). For example, two mutations in the *lacI* gene (the A nucleotide deletion at the position 221 and the G-to-T transversions at the position 754 of the *lacI* gene in the strains phe-lacI_117 and phe-lacI_105, respectively) were detected only in Phe^+^ mutants that emerged onto selective plates early, on days 3–5, but not later. The presence of strong positional effects in the occurrence of certain mutations particularly in growing cells is intriguing. In the light of the results presented in this study it is tempting to speculate that DNA replication complexes acting at different chromosomal locations in growing bacteria may contain different accessory factors which have dissimilar effects on the fidelity of DNA replication.

### Effect of DNA-binding Proteins on Mutagenesis

Nucleoid-associated proteins (NAPs) and other DNA binding proteins (e.g., various transcription factors) fold bacterial chromosome into higher-order structures and alter the level of gene expression [63], [64]. Mutations can occur during the chromosome replication and as a result of DNA repair synthesis carried out at the sites of DNA damage. It is possible that regional differences in chromosomal topology may cause unequal access of chromosomal regions to mutagenesis by influencing formation of DNA repair complexes and participation of specialized DNA polymerases in DNA synthesis.

Our current results demonstrate that the frequency of transposition of IS element IS*1411* also varies at different chromosomal positions of *P. putida* (Table 3). To avoid potentially deleterious effect of transposition to the host genome, the frequency of transposition in a cell is down-regulated both by transposon-encoded and host-encoded factors. Transposition may be regulated by controlling transposase TnpA expression (transcriptional, translational, and/or posttranslational control mechanisms) and also by factors that affect the transposition process itself [65]. So far, little is known about the regulation of transposition of IS*1411*. The results of our previous studies indicate that transposition of IS*1411* occurs through a circular intermediate [40]. We suppose that the process of circle formation and transposition of this IS element is replicative, since the copy of the element has always retained at its original location. The frequency of transposition of IS*1411* increases with time of starvation in *P. putida* lacking stationary-phase sigma factor RpoS, indicating that expression of some factor(s) which down regulate IS*1411* transposition activity require RpoS, and that activation of IS*1411* needs some late-starvation signal [62]. In the current study we have detected the insertions of IS*1411* into the *lacI* repressor gene both in early- and late-appearing mutants (Table 4 and Table S7). However, at certain chromosomal positions (e.g., in the strains phe-lacI_105 and phe-lacI_117 which exhibited the highest frequency of IS*1411* insertions) the transposition events were mostly detected among late-appearing mutants. The fact that the frequency of transposition of IS*1411* into the *lacI* gene varies at different chromosomal positions leads us to speculate that regional differences in nucleoid folding may also influence its transposition. It is known that the level of DNA supercoiling affects some transposition reactions [66], [67]. The level of DNA supercoiling is regulated by the combined activities of topoisomerases and NAPs [68]. It is well established that the superhelical density of DNA varies according to the growth conditions and that this change involves differential expression and DNA binding of NAPs [63], [64], [68]. Such spatiotemporal changes in DNA topology may influence transposition of IS*1411.*

### Concluding Remarks

Given the complexities of mechanisms of mutagenesis, none of the above-discussed mechanisms alone provides an explanation regarding the observed variation in the frequency of mutations at different chromosomal positions. In addition to the effects caused by the co-directional or head-on orientations of RNA polymerase and the replisome, the frequency of mutations at the distinct chromosomal sites might be affected by several other factors. In some cases (e.g., the occurrence of 1-bp deletions within the run of seven C-nucleotides and the preferred occurrence of CTGG insertions or deletions at the repeated sequence), an effect of DNA strand bias (leading or lagging strand replication) on the mutagenic processes was observed. Additionally, we cannot exclude the effect of the level of transcription. It is also noteworthy that certain mutational hot spots were detected only at particular chromosomal positions and especially in growing bacteria. Thus, it seems plausible that regional differences in chromosome structure and organization influence mutagenic processes in growing bacteria more strongly than previously assumed. At the same time, since the mutants continued to accumulate in starving populations of *P. putida*, some cells could still grow slowly and replicate their chromosome under the starvation conditions. Nevertheless, it is also possible that mutations in the chromosome of *P. putida* stationary-phase cells have mainly occurred during the course of DNA repair synthesis.

The fact that mutation frequency and spectrum of mutations vary across the bacterial chromosome could play an important role in divergence of bacterial populations in nature. Depending on the location of the potential target genes in the chromosome some mutational pathways may prevail over the others in the evolution of bacteria.

## Experimental Procedures

### Bacterial Strains, Plasmids and Media

Bacterial strains and plasmids used in this study are described in Table S1 and primers for DNA amplification in Table S2. Complete medium was Luria-Bertani (LB) medium [69], and minimal medium was M9 [70]. Solid medium contained 1.5% Difco agar. Casamino acids (CAA) and glucose were added to the minimal medium at final concentrations of 0.2% and 10 mM, respectively. Phenol minimal plates contained 2.5 mM phenol as a sole carbon and energy source. Antibiotics were added at the following final concentrations: for *E. coli*, ampicillin at 100 µg ml^−1^; for *P. putida*, tetracycline at 50 µg ml^−1^, carbenicillin at 1500 to 3000 µg ml^−1^, rifampicin 100 µg ml^−1^, and potassium tellurite at 70 µg ml^−1^; for both organisms, kanamycin at 50 µg ml^−1^. *E. coli* was incubated at 37°C and *P. putida* at 30°C. *E. coli* and *P. putida* were electrotransformed as described by Sharma and Schimke [71]. *E. coli* strains DH5α (Invitrogen), and CC118 λpir [72] were used for the DNA cloning procedures and HB101 [73] as a host for helper plasmid pRK2013 [74], necessary for the mobilization of non-conjugative plasmids.

### Construction of Test Systems Detecting Occurrence of Mutations in *P. putida* Chromosome

The assay systems for the detection of mutations in the chromosome of *P. putida*, based on the activation of the phenol monooxygenase gene *pheA*, enable bacteria to use phenol as a sole source of carbon and energy. One of the test systems (phe-lacI) was constructed for the detection of broad spectrum of mutations either inactivating the *lacI* repressor gene or altering the *lac* operator sequence which negatively controls the transcription of the phenol monooxygenase gene *pheA* from the P*_tac_* promoter. Another test system (pheA+C) was designed for the measurement of one specific mutation, deletion of one nucleotide within a run of seven C-nucleotides leading to the reversion of the reading frame of the *pheA* gene. Both test systems were randomly inserted into the chromosome of *P. putida* strain PaW85 [75], [76] within a mini-Tn*5* transposon.

For the construction of the phe-lacI test system, at first the DNA fragment containing the P*_tac_* promoter and *lacI* repressor gene was cut from the plasmid pBRlacItac [77] using the restrictase BamHI and inserted into pUC18NotKm to obtain plasmid pUC18NotlacI. The plasmid pUC18NotKm was constructed by inserting the Km-resistance gene from plasmid pUTmini-Tn*5* Km2 [78] within the 1430-bp Eco47III-generated DNA fragment into the DraI-cleaved plasmid pUC18Not [72]. The restriction enzyme DraI cleaves pUC18Not three times, once at the beginning of the β-lactamase gene *bla* and twice downstream from this gene. Thus, this strategy enabled us to replace the *bla* gene sequence in pUC18Not with the Km-resistance encoding gene. The Ecl136II- and EcoRI-generated DNA fragment containing the *pheBA* genes and IS element IS*1411* from the plasmid pEST1414 [29] was inserted into the Ecl136II- and EcoRI-cleaved plasmid pUC18NotlacI yielding the plasmid pUC18NotlacIpheBA. Then, pUC18NotlacIpheBA was cleaved with NotI to insert the *lacI*-P*_tac_*-*pheBA* cassette from pUC18NotlacIpheBA into the NotI-cleaved mini-Tn*5* delivery plasmid pJMT6 [79], resulting in the plasmid pUTlacIpheBA.

To construct the other mutation detection system pheA+C for the monitoring occurrence of 1-bp deletions, the *pheA* coding sequence was altered by inserting a single C nucleotide at position 221 relative to the translational initiator codon of this gene. The nucleotide insertion site already contained six C nucleotides. The frameshift mutation was performed by PCR amplification of the segment of the *pheA* gene from the plasmid pPU1930 [80] with primer pheABamei and the mutant primer pheAvi+1 (Table S2). The amplified DNA fragment was subcloned into the pBluescript KS(+) EcoRV site to obtain pKSpheA+C. The +1 frameshift mutation was verified by DNA sequencing. The mutated DNA fragment was thereafter inserted as XbaI- and AviII- generated fragment from pKSpheA+C into pPU1930 by replacing the original *pheA* sequence located between the XbaI and AviII sites to generate the plasmid pPUpheA+C. Thereafter, we inserted the constitutively expressed P*_GC_* promoter and the *pheA* allele with +1 frameshift as the Ecl126II- and PvuII-generated fragment from pPUpheA+C containing into the Ecl136II-cleaved pUC18NotKm to obtain the plasmid pUC18NotpheA+C. Finally, the plasmid pUC18NotpheA+C was cleaved with NotI to insert the P*_GC_-pheA*+C cassette into the NotI-cleaved mini-Tn*5* delivery plasmid pJMT6, yielding the plasmid pUTpheA+C.

The mutation detection system-carrying plasmids pUTlacIpheBA and pUTpheA+C, which do not replicate in hosts other than *E. coli* strain CC118λpir, were conjugatively transferred into *P. putida* strain PaW85 by using the helper plasmid pRK2013 [74]. Transconjugants carrying random insertions of the test system within mini-Tn*5* in the chromosome of *P. putida* were isolated. Integration of whole delivery plasmid into *P. putida* chromosome was excluded by testing transconjugants for resistance to carbenicillin. Only those sensitive to carbenicillin represented a true transposition event. Additionally, strains either carrying the phe-lacI or pheA+C test system in the chromosome were confirmed by PCR analysis.

### Construction of Assay System for the Measurement of Effect of Transcription of the Mutation Target Gene on Mutation Frequency

To study the effect of the level of transcription of the mutation target gene on mutation frequency, we modified the pheA+C test system by placing the transcription of the mutated *pheA* allele under the control of IPTG-inducible P*_tac_* promoter. At first, the Ecl136II- and PvuII-generated DNA fragment from pPUpheA+C containing the P*_GC_-pheA*+C cassette was inserted into Ecl136II-cleaved pUC18NotlacI containing the P*_tac_* promoter and *lacI* repressor gene, yielding the plasmid pUC18NotlacIpheA+C. Thereafter, pUC18NotlacIpheA+C was cleaved with NotI to insert the *lacI*-P*_tac_*-*pheA*+C cassette from pUC18NotlacIpheA+C into the NotI-cleaved mini-Tn*5* delivery plasmid pJMT6 to obtain the plasmid pUTlacIpheA+C.

### Arbitrary PCR

To identify the location of the mutation detection system inserted randomly into *P. putida* PaW85 chromosome within mini-Tn*5* in various phe-lacI and pheA+C strains, arbitrary PCR and DNA sequencing were performed. PCR products were generated by two rounds of amplifications as described elsewhere [81]. In all cases, the chromosomal location of the test system was determined twice, by identifying the mini-Tn5-flanking chromosomal DNA sequences at both sides. Such double check ensured that the insertion sites were correctly determined and demonstrated that the insertion of the mini-Tn into the chromosome did not cause genetic rearrangements at the flanking DNA. In the first round of PCR, primers ARBtel1, ARBpheA1 or pheAvaljasARB1 and arbitrary primers ARB6 or ARB-cggca were used. Second-round PCR was performed with primers ARBtel2, ARBpheA2 or pheAvaljasARB2 and arbitrary primer ARB2. DNA sequencing of the PCR products with the primers ARBtel2, ARBpheA2 or pheAvaljasARB2 was performed by using the BigDye Terminator v3.1 Cycle Terminator kit. The DNA sequencing reactions were analyzed with the Applied Biosystems 3730×l DNA sequencer.

### Comparison of Mutation Frequency in *P. putida* Strains Carrying the phe-lacI or pheA+C Test System in Different Chromosomal Locations

Conditions for the isolation of phenol-degrading Phe^+^ mutants were the same as those described in our previous study when we isolated Phe^+^ revertants which occurred due to point mutations [36], [82]. About 1×10^7^ to 4×10^7^ cells of the tester strains derived from independent cultures that were grown overnight in liquid M9 medium containing glucose and CAA were plated onto phenol-minimal plates. Independent cultures of the *P. putida* tester strains were generated by growing cells to late logarithmic growth phase in M9 medium containing glucose and CAA, diluting this culture by 10^5^ into fresh glucose and CAA-containing M9 medium, dispensing 2-ml aliquots into test tubes and allowing cells to reach saturation by growing cells for 18–20 h. The cells of the tester strains were mixed with approximately 1×10^9^ scavenger cells derived from the wild-type *P. putida* strain PaW85.

We have previously shown with plasmidial test systems [29], [36] that Phe^+^ colonies appearing on phenol minimal plates on day 2 contained mutations that occurred before the plating in a growing culture, whereas colonies that emerged on selective plates on day 3 and later contained mutations that occurred after the cells were plated. Latter were called stationary-phase mutations. We expected that the growth of the Phe^+^ mutants detected with chromosomal test systems could be slower compared to that measured previously for the Phe^+^ mutants detected with plasmidial assay systems. In order to find out how long it would take for the appearance of Phe^+^ colonies onto selective plates in our assays (i.e., to distinguish mutations occurring in growing cultures from those occurring after plating), we carried out reconstruction experiments by mixing about 100–200 cells of individual Phe^+^ mutants picked up from selective plates on different days (on days 4 to 7) with 5×10^8^ scavenger cells not containing the *pheA* gene and monitored the appearance of Phe^+^ colonies on phenol minimal plates.

The frequency of Phe^+^ mutations occurring in stationary-phase populations was determined per viable cells on the selective plates. The viability of the tester cells during incubation under long-term carbon starvation conditions was determined on the same plates that were used for the isolation of Phe^+^ mutants. Small plugs were cut from the plates avoiding Phe^+^ colonies. Bacteria from these plugs were suspended in M9 buffer, and dilutions were plated onto LB plates containing potassium tellurite to determine the number of colony forming units of the tester strain in starving populations. In the case of all tester strains studied, the viability of bacteria during the starvation did not decrease significantly.

The frequency of occurrence of Rif^r^ mutations in growing cells of different *P. putida* strains was performed as previously described [83] except that the *P. putida* cultures were grown in M9 medium supplemented with glucose and CAA. Emergence of Rif^r^ colonies was counted after 48 h of incubation to give enough time to grow up also for mutants expressing milder Rif^r^ phenotype. The frequency of Rif^r^ mutations was determined at least in 45 independent cultures for each *P. putida* strain. The median value for Rif^r^ mutants per 1×10^9^ cells was calculated by using the Lea-Coulson method of the median [84].

### DNA Sequence Analysis of the Phe^+^ mutants

To characterize the spectrum of mutations in Phe^+^ mutants isolated with the phe-lacI test system, we PCR-amplified the *lacI* gene and the LacI operator sequence by using the primers lacopRev and lacIloppsisse1. The same primers and lacI3, lacI3out and lacIOc2 primers were used for DNA sequencing by the above-described methodology. BioEdit and ClustalW2 programs were used for sequence comparison.

### Statistical Analysis of the Results

The factorial ANOVA method and *post-hoc* Tukey HSD test were used to assess the variability of data in experiments comparing mutation frequency in different phe-lacI, pheA+C and Ptac-pheA+C strains. To obtain normal distribution, data were transformed to common logarithm, if it was necessary. To avoid taking common logarithm from zero, “1” was added for all data. For statistical tests the significance level was set at *P*<0.05. The calculations were performed using Statistica 10 software. The nonparametric Mann-Whitney U test was used for estimation of dependency of mutant frequency on the orientation of the mutational target gene. We grouped *P. putida* strains according to the direction of the target gene to the direction of movement of replisome. The group one contained the means of mutant frequency of the strains with co-directional and the group two that of the strains with head-on orientations of transcription of the target gene and movement of replisome.

The statistical significance of differences between the spectra of Phe^+^ mutations in strains carrying the phe-lacI test system in various chromosomal locations was determined by using the hypergeometric test algorithm described in Adams and Skopek [41] and Piegorsch and Bailer [85]. The software for performing the test [86] is available at http://www.ibiblio.org/dnam/des_hypg.htm. All *P* values were based on 30000 iterations. A *P*-value of <0.05 means that the spectra are different in a pairwise comparison, but since 6 mutational spectra were compared, a Bonferroni correction for multiple comparisons with a corrected significance level of 0.003 (0.05/5×6×0.5) should be applied. In pairwise comparison of individual sites in separate spectra the chi-square test of independence was performed by using a software program for statistical analyses (Statgraphics Centurion XV; Statpoint Inc.) and the significance level was set at *P*<0.05.

## Supporting Information

Figure S1 **Western blot analysis of crude cell lysates prepared from** ***P. putida*** **strains using polyclonal anti-PheA antibodies.** Cells were grown in LB liquid medium to optical density A_580_ = 0.9. Twenty micrograms of crude cell lysates were analysed. *P. putida* strain PaW85 has been used as a negative control.
(DOC)

Figure S2 **Effect of IPTG on the expression of phenol monooxygenase gene** ***pheA*** **in** ***P. putida*** **carrying the Ptac-pheA+C test system.** Western blot analysis of crude cell lysates prepared from *P. putida* strain PaW85 (negative control) and Phe^+^ revertant of *P. putida* strain Ptac-pheA+C_2 by using polyclonal anti-PheA antibodies. 50 micrograms of crude cell lysates were analyzed. **A.** PheA expression in growing cultures: lane 1, size marker; lane 2, PaW85; lane 3, PaW85+ IPTG; lane 4, Ptac-pheA+C_2 Phe^+^; lane 5, Ptac-pheA+C_2Phe^+^ + IPTG. Cells were grown in M9 minimal medium in the presence of glucose and CAA. **B.** PheA expression in stationary-phase cells: lane 1, size marker; lane 2, PaW85; lane 3, PaW85+ IPTG; lanes 4 and 6, Ptac-pheA+C_2 Phe^+^; lanes 5 and 7, Ptac-pheA+C_2Phe^+^ + IPTG. Bacteria were incubated for 3 days on M9 minimal agar plates either in the presence of glucose (lanes 2–5) or in the absence of any carbon source (lanes 6 and 7).
(DOC)

Table S1 **Bacterial strains and plasmids used in this study.**
(DOC)

Table S2 **Oligonucleotides used in this study.**
(DOC)

Table S3 **Location of the phe-lacI test system in** ***P. putida*** **PaW85 chromosome.**
(DOC)

Table S4 **Location of the pheA+C test system in** ***P. putida*** **PaW85 chromosome.**
(DOC)

Table S5 **The frequency of Rif^r^ mutants in** ***P. putida*** **strains carrying the phe-lacI test system at different chromosomal locations.**
(DOC)

Table S6 **The frequency of Rif^r^ mutants in** ***P. putida*** **strains carrying the pheA+C test system on different chromosomal locations.**
(DOC)

Table S7 **Location of the Ptac-pheA+C test system in** ***P. putida*** **PaW85 chromosome.**
(DOC)

Table S8 **Comparison of mutational spectra in strains carrying the phe-lacI test system at different chromosomal locations.**
(DOC)

Table S9 **The results of pairwise comparison of mutation frequency at individual sites obtained with chi-square test. Only the results with** ***P*** **<0.005 are shown.**
(DOC)

Table S10 **Spectrum of Phe^+^ mutations on separate days in** ***P. putida*** **strains carrying the phe-lacI test system in various chromosomal positions.**
(DOC)

Table S11 **Comparison of mutational spectra on different time periods in strains carrying the phe-lacI test system at various chromosomal locations.**
(DOC)

Table S12 **The results of pairwise comparison of mutation frequency on different time periods at individual sites obtained with chi-square test. Only the results with** ***P*** **<0.005 are shown.**
(DOC)
