# Supplementary material for: Mutation Frequency and Spectrum of Mutations Vary at Different Chromosomal Positions of Pseudomonas putida
Source: PLoS One. 2012 Oct 31;7(10):e48511. doi: 10.1371/journal.pone.0048511 (PMC3485313; doi:10.1371/journal.pone.0048511)
Supplement: Figure S1 — Western blot analysis of crude cell lysates prepared from P. putida strains using polyclonal anti-PheA antibodies. Cells were grown in LB liquid medium to optical density A580 = 0.9. Twenty micrograms of crude cell lysates were analysed. P. putida strain PaW85 has been used as a negative control. (DOC) [file pone.0048511.s001.doc]

Figure S1


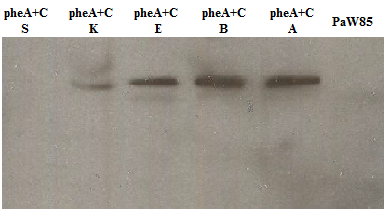


Western blot analysis of crude cell lysates prepared from *P. putida* strains using polyclonal anti-PheA antibodies. Cells were grown in LB liquid medium to optical density A_580_ = 0.9. Twenty micrograms of crude cell lysates were analysed. *P. putida* strain PaW85 has been used as a negative control.
