# Supplementary material for: Mutation Frequency and Spectrum of Mutations Vary at Different Chromosomal Positions of Pseudomonas putida
Source: PLoS One. 2012 Oct 31;7(10):e48511. doi: 10.1371/journal.pone.0048511 (PMC3485313; doi:10.1371/journal.pone.0048511)
Supplement: Figure S2 — Effect of IPTG on the expression of phenol monooxygenase gene pheA in P. putida carrying the Ptac-pheA+C test system. Western blot analysis of crude cell lysates prepared from P. putida strain PaW85 (negative control) and Phe+ revertant of P. putida strain Ptac-pheA+C_2 by using polyclonal anti-PheA antibodies. 50 micrograms of crude cell lysates were analyzed. A. PheA expression in growing cultures: lane 1, size marker; lane 2, PaW85; lane 3, PaW85+ IPTG; lane 4, Ptac-pheA+C_2 Phe+; lane 5, Ptac-pheA+C_2Phe+ + IPTG. Cells were grown in M9 minimal medium in the presence of glucose and CAA. B. PheA expression in stationary-phase cells: lane 1, size marker; lane 2, PaW85; lane 3, PaW85+ IPTG; lanes 4 and 6, Ptac-pheA+C_2 Phe+; lanes 5 and 7, Ptac-pheA+C_2Phe+ + IPTG. Bacteria were incubated for 3 days on M9 minimal agar plates either in the presence of glucose (lanes 2–5) or in the absence of any carbon source (lanes 6 and 7). (DOC) [file pone.0048511.s002.doc]

Figure S2

Effect of IPTG on the expression of phenol monooxygenase gene *pheA* in *P. putida* carrying the Ptac-pheA+C test system. Western blot analysis of crude cell lysates prepared from *P. putida* strain PaW85 (negative control) and Phe^+^ revertant of *P. putida* strain Ptac-pheA+C_2 by using polyclonal anti-PheA antibodies. 50 micrograms of crude cell lysates were analyzed. **A.** PheA expression in growing cultures: lane 1, size marker; lane 2, PaW85; lane 3, PaW85 + IPTG; lane 4, Ptac-pheA+C_2 Phe^+^; lane 5, Ptac-pheA+C_2Phe^+^ + IPTG. Cells were grown in M9 minimal medium in the presence of glucose and CAA. **B.** PheA expression in stationary-phase cells: lane 1, size marker; lane 2, PaW85; lane 3, PaW85 + IPTG; lanes 4 and 6, Ptac-pheA+C_2 Phe^+^; lanes 5 and 7, Ptac-pheA+C_2Phe^+^ + IPTG. Bacteria were incubated for 3 days on M9 minimal agar plates either in the presence of glucose (lanes 2-5) or in the absence of any carbon source (lanes 6 and 7).
