# Supplementary material for: Mutation Frequency and Spectrum of Mutations Vary at Different Chromosomal Positions of Pseudomonas putida
Source: PLoS One. 2012 Oct 31;7(10):e48511. doi: 10.1371/journal.pone.0048511 (PMC3485313; doi:10.1371/journal.pone.0048511)
Supplement: Table S1 — Bacterial strains and plasmids used in this study. (DOC) [file pone.0048511.s003.doc]

**Supporting tables**

Table S1. Bacterial strains and plasmids used in this study

| *E. coli* strains | Genotype/description | Source/reference |
| --- | --- | --- |
| DH5α | *supE44* Δ*lacU169 recA1 endA1 hsdR17 thi-1 gyrA96 relA1* | Invitrogen |
| HB101 | *subE44 subF58 hsdS3*(r_B_^-^ m_B_^-^) *recA13 ara-14 proA2 lacY1 galK2 rpsL20 xyl-5 mtl-1* | [1] |
| CC118 λpir | Δ*(ara-leu) araD* Δ*lacX74 galE galK phoA20 thi-1 rpsE rpoB argE (*Am*) recA1 λpir*  phage lysogen | [2] |
| *P. putida* strains |  |  |
| PaW85 | Wild type , same as KT2440 | [3,4] |
| phe-lacI_1 | PaW85, phe-lacI test system in chromosome, unknown location | This study |
| phe-lacI_5 | PaW85, phe-lacI test system in chromosomal locus PP0340 | This study |
| phe-lacI_6 | PaW85, phe-lacI test system in chromosomal locus PP1186 | This study |
| phe-lacI_7 | PaW85, phe-lacI test system in chromosomal locus PP2818 | This study |
| phe-lacI_8 | PaW85, phe-lacI test system in chromosome, unknown location | This study |
| phe-lacI_10 | PaW85, phe-lacI test system in chromosomal locus PP4644 | This study |
| phe-lacI_11 | PaW85, phe-lacI test system in chromosomal locus PP2332 | This study |
| phe-lacI_12 | PaW85, phe-lacI test system in chromosome, unknown location | This study |
| phe-lacI_13 | PaW85, phe-lacI test system in chromosomal locus PP1577 | This study |
| phe-lacI_14 | PaW85, phe-lacI test system in chromosomal locus PP1628 | This study |
| phe-lacI_16 | PaW85, phe-lacI test system in chromosomal locus PP4624 | This study |
| phe-lacI_18 | PaW85, phe-lacI test system in chromosomal locus PP3728 | This study |
| phe-lacI_19 | PaW85, phe-lacI test system in chromosomal locus PP2764 | This study |
| phe-lacI_20 | PaW85, phe-lacI test system in chromosomal locus PP3379 | This study |
| phe-lacI_23 | PaW85, phe-lacI test system in chromosomal locus PP2448 | This study |
| phe-lacI_24 | PaW85, phe-lacI test system in chromosomal locus PP0052 | This study |
| phe-lacI_25 | PaW85, phe-lacI test system in chromosomal locus PP2556 | This study |
| phe-lacI_26 | PaW85, phe-lacI test system in chromosomal locus PP2436 | This study |
| phe-lacI_30 | PaW85, phe-lacI test system in chromosomal locus PP2316 | This study |
| phe-lacI_31 | PaW85, phe-lacI test system in chromosomal locus PP4050 | This study |
| phe-lacI_105 | PaW85, phe-lacI test system in chromosomal locus PP2501 | This study |
| phe-lacI_110 | PaW85, phe-lacI test system in chromosomal locus PP0532 | This study |
| phe-lacI_115 | PaW85, phe-lacI test system in chromosomal locus PP3579 | This study |
| phe-lacI_117 | PaW85, phe-lacI test system in chromosomal locus PP4158 | This study |
| pheA+C_A | PaW85, pheA+C test system in chromosomal locus PP2387 | This study |
| pheA+C_B | PaW85, pheA+C test system in chromosomal locus PP2981 | This study |
| pheA+C_D | PaW85, pheA+C test system in chromosomal locus PP3927 | This study |
| pheA+C_E | PaW85, pheA+C test system in chromosomal locus PP3820 | This study |
| pheA+C_F | PaW85, pheA+C test system in chromosomal locus PP2839 | This study |
| pheA+C_G | PaW85, pheA+C test system in chromosomal locus PP2316 | This study |
| pheA+C_I | PaW85, pheA+C test system in chromosomal locus PP1666 | This study |
| pheA+C_J | PaW85, pheA+C test system in chromosomal locus PP1047 | This study |
| pheA+C_K | PaW85, pheA+C test system in chromosomal locus PP1446 | This study |
| pheA+C_N | PaW85, pheA+C test system in chromosomal locus PP3200 | This study |
| pheA+C_O | PaW85, pheA+C test system in chromosomal locus PP3831 | This study |
| pheA+C_P | PaW85, pheA+C test system in chromosomal locus PP2828 | This study |
| pheA+C_Q | PaW85, pheA+C test system in chromosomal locus PP3804 | This study |
| pheA+C_S | PaW85, pheA+C test system in chromosomal locus PP2981 | This study |
| Ptac-pheA+C_2 | PaW85, Ptac-pheA+C test system in chromosomal locus PP1517 | This study |
| Ptac-pheA+C_3 | PaW85, Ptac-pheA+C test system in chromosomal locus PP2094 | This study |
| Ptac-pheA+C_4 | PaW85, Ptac-pheA+C test system in chromosomal locus PP4517 | This study |
| Ptac-pheA+C_13 | PaW85, PtacpheA+C test system in chromosomal locus PP3007 | This study |
| Plasmids | Genotype/description | Source/reference |
| pEST1414 | Plasmid pAYC32 carrying the promoterless *pheBA* operon | [5] |
| pBRlacItac | P*_tac_* promoter and *lacI*^q^ repressor in plasmid pBR322 | [6] |
| pUTmini-Tn5 Km2 | Delivery plasmid for mini-Tn*5* Km (Amp^r^, Km^r^) | [7] |
| pJMT6 | Delivery plasmid for mini-Tn*5* Tel (Amp^r^, Tel^r^) | [8] |
| pRK2013 | Helper plasmid for conjugal transfer of mini-Tn-carrying plasmids (Km^r^) | [9] |
| pUC18Not | Cloning vector (Amp^r^) | [2] |
| pUC18NotKm | pUC18 derivative (Km^r^) carrying Km^r^ gene from pUTmini-Tn5 Km2 instead *bla* gene  cloned as 1430-bp Eco47III fragment into DraI-cleaved pUC18Not | This study |
| pUC18NotlacI | pUC18NotKm with P*_tac_* promoter and *lacI*^q^ repressor cloned from pBRlacItac  within BamHI fragment in BamHI-cleaved vector (Km^r^) | This study |
| pUC18NotlacIpheBA | pUC18NotlacI with *pheBA* genes and IS*1411*-containing Ecl136II-EcoRI fragment  from pEST1411 inserted into Ecl136II- and EcoRI-cleaved vector (Km^r^) | This study |
| pUTlacIpheBA | mini-Tn5 delivery plasmid pJMT6 containing the *lacI*-P*_tac_*-*pheBA* cassette cloned  as NotI fragment from pUC18NotlacIpheBA into NotI-cleaved vector (Amp^r^, Tel^r^) | This study |
| pPU1930 | *pheA* gene with constitutively expressed P*_GC_* promoter in plasmid pUC18 (Ap^r^) | [10] |
| pBluescript KS(+) | Cloning vector (Ap^r^) | Stratagene |
| pKSpheA+C | pBluescript KS(+) containing PCR-amplified *pheA* sequence with C-nucleotide  insertion cloned into EcoRV-cleaved vector | This study |
| pPUpheA+C | Mutated *pheA* sequence cloned as XbaI-and AviI-generated fragment from  pKSpheA+C into pPU1930 to replace the original *pheA* sequence (Ap^r^) | This study |
| pUC18NotpheA+C | pUC18NotKm with P*_GC_*-*pheA*+C cassette cloned from pPUpheA+C within Ecl136II-  and PvuII-generated fragment into Ecl136II-cleaved vector (Km^r^) | This study |
| pUTpheA+C | mini-Tn5 delivery plasmid pJMT6 containing the P*_GC_*-*pheA*+C cassette cloned  as NotI fragment from pUC18NotpheA+C into NotI-cleaved vector (Amp^r^, Tel^r^) | This study |
| pUC18NotlacIpheA+C | P*_GC_*-*pheA*+C cassette within Ecl136II-and PvuII-generated fragment from pPUpheA+C  inserted into Ecl136II-cleaved pUC18NotlacI | This study |
| pUTlacIpheA+C | mini-Tn5 delivery plasmid pJMT6 containing the *lacI*-P*_tac_*-P*_GC_*-*pheA*+C cassette  cloned as NotI fragment from pUC18NotlacIpheA+C into NotI-cleaved vector | This study |

**References**

1. Boyer HW, Roulland-Dussoix D (1969) A complementation analysis of the restriction and modification of DNA in *Escherichia coli*. J Mol Biol 41: 459-472.

2. Herrero M, de Lorenzo V, Timmis KN (1990) Transposon vectors containing non-antibiotic resistance selection markers for cloning and stable chromosomal insertion of foreign genes in gram-negative bacteria. J Bacteriol 172: 6557-6567.

3. Bayley SA, Duggleby CJ, Worsey MJ, Williams PA, Hardy KG, et al. (1977) Two modes of loss of the Tol function from *Pseudomonas putida* mt-2. Mol Gen Genet 154: 203-204.

4. Regenhardt D, Heuer H, Heim S, Fernandez DU, Strompl C, et al. (2002) Pedigree and taxonomic credentials of *Pseudomonas putida* strain KT2440. Environ Microbiol 4: 912-915.

5. Kasak L, Hõrak R, Kivisaar M (1997) Promoter-creating mutations in *Pseudomonas putida*: a model system for the study of mutation in starving bacteria. Proc Natl Acad Sci U S A 94: 3134-3139.

6. Ojangu EL, Tover A, Teras R, Kivisaar M (2000) Effects of combination of different -10 hexamers and downstream sequences on stationary-phase-specific sigma factor sigma(S)-dependent transcription in *Pseudomonas putida*. J Bacteriol 182: 6707-6713.

7. de Lorenzo V, Herrero M, Jakubzik U, Timmis KN (1990) Mini-Tn*5* transposon derivatives for insertion mutagenesis, promoter probing, and chromosomal insertion of cloned DNA in gram-negative eubacteria. J Bacteriol 172: 6568-6572.

8. Sanchez-Romero JM, Diaz-Orejas R, De Lorenzo V (1998) Resistance to tellurite as a selection marker for genetic manipulations of *Pseudomonas* strains. Appl Environ Microbiol 64: 4040-4046.

9. Figurski DH, Helinski DR (1979) Replication of an origin-containing derivative of plasmid RK2 dependent on a plasmid function provided in trans. Proc Natl Acad Sci U S A 76: 1648-1652.

10. Nurk A, Kasak L, Kivisaar M (1991) Sequence of the gene (*pheA*) encoding phenol monooxygenase from *Pseudomonas* sp. EST1001: expression in *Escherichia coli* and *Pseudomonas putida*. Gene 102: 13-18.
