# Supplementary material for: Mutation Frequency and Spectrum of Mutations Vary at Different Chromosomal Positions of Pseudomonas putida
Source: PLoS One. 2012 Oct 31;7(10):e48511. doi: 10.1371/journal.pone.0048511 (PMC3485313; doi:10.1371/journal.pone.0048511)
Supplement: Table S2 — Oligonucleotides used in this study. (DOC) [file pone.0048511.s004.doc]

**Table S2. Oligonucleotides used in this study**

| Purpose | Oligonucleotide **designation** | **Oligonucleotide sequence (complementary region)** |
| --- | --- | --- |
| Construction of chromosomal  pheA+C test system | pheABamei | 5´-AAGGCGGCTCCCGTAAGACA-3´ (positions -40 to -22 in relation to  the translation initiator codon of the *pheA* gene) |
|  | pheAvi+1 | 5´-ATACGCGTGCCCCCCCATAA-3´ (positions 214 to 232 in the *pheA*  gene, contains extra C nucleotide at position 221) |
| Arbitrary PCR and sequencing | ARBtel1 | 5´-GGGTGCAGAGCCATAAGC-3´ (positions 938 to 955 in the *telB* gene) |
|  | ARBpheA1 | 5´-AACTACGTTGGTCTCATTTGG-3´ (positions 102 to 82 in *pheA*) |
|  | pheAvaljasARB1 | 5´-TCTACCTGAGAGTGATCATATG-3´ (positions 3341 to 3362  downstream from the *pheA* translation initiator codon, outside *pheA* gene) |
|  | ARB6 | 5´-GGCCACGCGTCGACTAGTACNNNNNNNNNNACGCC-3´ (arbitrary  primer; *P. putida* genome contains ACGCC linker sequence 12050 times) |
|  | ARB-cggca | 5´-GGCCACGCGTCGACTAGTACNNNNNNNNNNCGGCA-3´ (arbitrary  primer; *P. putida* genome contains CGGCA linker sequence 16341 times) |
|  | ARBtel2 | 5´-GGGTGCAGAGCCATAAGC-3´ (positions 956 to 973 in *telB*) |
|  | ARBpheA2 | 5´-TCAAGATTATCATTACGCTGTG-3´ (positions 29 to 8 in *pheA*) |
|  | pheAvaljasARB2 | 5´-TGAGCTTCTCGTCAGTCGTC-3´ (positions 3372 to 3391  downstream from the *pheA* translation initiator codon, outside *pheA* gene) |
|  | ARB2 | 5´-GGCCACGCGTCGACTAGTAC-3´ (complementary to the sequence of  PCR products obtained in the first round of arbitrary PCR) |
| Sequence analysis of Phe^+^ mutants  obtained with phe-lacI test system | lacopRev | 5´-GATCCTCTAGAGTCGACCTG-3´ (positions -420 to -401 in relation to  the translation initiator codon of the *lacI* gene) |
|  | lacI3 | 5´-CATCGCAGTGGGAACGATG-3´ (positions 726 to 707 in *lacI* gene) |
|  | lacI3out | 5´-ATTCAGCCGATAGCGGAACGG-3´ (positions 628 to 648 in *lacI* gene) |
|  | lacIOc2 | 5´-TGCCCGCCAGTTGTTGTGCCA-3´ (positions 175 to 154 in *lacI* gene) |
