# Supplementary material for: Mutation Frequency and Spectrum of Mutations Vary at Different Chromosomal Positions of Pseudomonas putida
Source: PLoS One. 2012 Oct 31;7(10):e48511. doi: 10.1371/journal.pone.0048511 (PMC3485313; doi:10.1371/journal.pone.0048511)
Supplement: Table S3 — Location of the phe-lacI test system in P. putida PaW85 chromosome. (DOC) [file pone.0048511.s005.doc]

**Table S3. Location of the phe-lacI test system in *P. putida* PaW85 chromosome**

| Strain | Locus ID | Gene name | Protein name | Insertion position along  the genome^a^ |
| --- | --- | --- | --- | --- |
| phe-lacI_5 | PP0340 | *glnE* | Glutamate-ammonia-ligase adenyl transferase | 2,165,498 → |
| phe-lacI_6 | PP1186 | *phoP* | DNA-binding response regulator PhoP | 1,361,115 → |
| phe-lacI_7 | PP2818 | *mexD* | Multidrug efflux RND transporter MexD | 3,217,174 ← |
| phe-lacI_10 | PP4644 | *radA* | DNA repair protein RadA | 5,270,161 → |
| phe-lacI_11 | PP2332 |  | Conserved hypothetical protein | 2,660,499 ← |
| phe-lacI_13 | PP1577 |  | Lambda family phage tail tape measure protein | 1,767,770 → |
| phe-lacI_14 | PP1628 |  | CinA domain-containing protein | 1,826,925 → |
| phe-lacI_16 | PP4624 |  | Hydrolase, alpha/beta fold family hydrolase | 5,249,456 → |
| phe-lacI_18 | PP3728 |  | Multi-sensor hybrid histidine kinase | 4,255,078 ← |
| phe-lacI_19 | PP2764 | *msuE* | NADH-dependent FMN reductase MsuE | 3,148,466 → |
| phe-lacI_20 | PP3379 | *kguK* | Xylose isomerase | 3,824,233 ← |
| phe-lacI_23 | PP2448 |  | FAD dependent oxidoreductase | 2.795,512 ← |
| phe-lacI_24 | PP0052 |  | Beta-lactamase domain protein | 61,179 → |
| phe-lacI_25 | PP2556 |  | Chromate transporter | 2,904,590 ← |
| phe-lacI_26 | PP2436 |  | LysR family transcriptional regulator | 2,783,298 ← |
| phe-lacI_30 | PP2316 |  | ABC transporter, permease protein, putative | 2,645,281 ← |
| phe-lacI_31 | PP4050 | *glgA* | Glycogen synthase/ alpha amylase family protein | 4,565,560 → |
| phe-lacI_105 | PP2501 |  | Phage integrase | 2,848,051 → |
| phe-lacI_110 | PP0532 |  | Esterase | 617,971 → |
| phe-lacI_115 | PP3579 |  | Conserved hypothetical protein | 4,061,029 → |
| phe-lacI_117 | PP4158 | *kdpD* | Osmosensitive K^+^ channel signal transduction  histidine kinase KdpD | 4,699,697 ← |

^a^Arrow shows the orientation of the mutational target gene *lacI* along the genome.
