# Supplementary material for: Mutation Frequency and Spectrum of Mutations Vary at Different Chromosomal Positions of Pseudomonas putida
Source: PLoS One. 2012 Oct 31;7(10):e48511. doi: 10.1371/journal.pone.0048511 (PMC3485313; doi:10.1371/journal.pone.0048511)
Supplement: Table S4 — Location of the pheA+C test system in P. putida PaW85 chromosome. (DOC) [file pone.0048511.s006.doc]

**Table S4. Location of the pheA+C test system in *P. putida* PaW85 chromosome**

| Strain | Locus ID | Gene name | Protein name | Insertion position along  the genome^a^ |
| --- | --- | --- | --- | --- |
| pheA+C_A | PP2387 |  | Conserved hypothetical protein | 2,726,511 ← |
| pheA+C_B | PP2981 | *tnpS* | Tn*4652*, cointegrate resolution protein S | 3,379,549 ← |
| pheA+C_D | PP3927 |  | Methyltransferase type 12 | 4,432,283 → |
| pheA+C_E | PP3820 |  | Group II intron-encoding maturase | 4,348,439 → |
| pheA+C_F | PP2839 |  | DEAD_2 domain protein | 3,243,877 → |
| pheA+C_G | PP2316 |  | ABC transporter, permease protein | 2,646,896 ← |
| pheA+C_I | PP1666 |  | Conserved hypothetical protein | 1,862,490 → |
| pheA+C_J | PP1047 | *xcpR* | Type II secretion system protein E | 1,197,970 → |
| pheA+C_K | PP1446 |  | TonB-dependent receptor | 1,649,283 → |
| pheA+C_N | PP3200 |  | Conserved hypothetical protein | 3,630,109 → |
| pheA+C_O | PP3831 |  | DNA topoisomerase I | 4,357,046 → |
| pheA+C_P | PP2828 |  | Conserved hypothetical protein | 3,227,879 → |
| pheA+C_Q | PP3804 |  | Conserved hypothetical protein | 4,332,689 → |
| pheA+C_S | PP2981 | *tnpS* | Tn*4652*, cointegrate resolution protein S | 3,379,441 → |

^a^Arrow shows the orientation of the mutational target gene *pheA* along the genome.
