# Supplementary material for: Mutation Frequency and Spectrum of Mutations Vary at Different Chromosomal Positions of Pseudomonas putida
Source: PLoS One. 2012 Oct 31;7(10):e48511. doi: 10.1371/journal.pone.0048511 (PMC3485313; doi:10.1371/journal.pone.0048511)
Supplement: Table S5 — The frequency of Rifr mutants in P. putida strains carrying the phe-lacI test system at different chromosomal locations. (DOC) [file pone.0048511.s007.doc]

**Table S5.** The frequency of Rif^r^ mutants in *P. putida* strains carrying the phe-lacI test system at different chromosomal locations

| **Strain^a^** | **Rif^r^ mutants per 10^9^ cells^b^** |
| --- | --- |
| **phe-lacI_16** | **14.597 (2.82)** |
| **phe-lacI_30** | **14.21 (3.06)** |
| phe-lacI_20 | 14.26 (3.18) |
| phe-lacI_5 | 12.74 (2.48) |
| phe-lacI_19 | 11.18 (2.38) |
| **phe-lacI_31** | **12.32 (3.64)** |
| **phe-lacI_25** | **11.42 (2.79)** |
| phe-lacI_18 | 11.08 (3.21) |
| **phe-lacI_115** | **10.1 (2.75)** |
| phe-lacI_110 | 9.36 (2.15) |
| phe-lacI_105 | 8.77 (1.8) |
| phe-lacI_ 117 | 8.08 (2.16) |

^a^ Strains carrying the mutational target gene opposite to the direction of the movement of replisome in the chromosome are indicated in bold.

^b^Average numbers of Rif^r^ mutants per 1 x 10^9^ cells with 95% confidence intervals are shown. At least 45 independent cultures were examined for each strain.
