# Supplementary material for: Mutation Frequency and Spectrum of Mutations Vary at Different Chromosomal Positions of Pseudomonas putida
Source: PLoS One. 2012 Oct 31;7(10):e48511. doi: 10.1371/journal.pone.0048511 (PMC3485313; doi:10.1371/journal.pone.0048511)
Supplement: Table S6 — The frequency of Rifr mutants in P. putida strains carrying the pheA+C test system on different chromosomal locations. (DOC) [file pone.0048511.s008.doc]

**Table** **S6**. The frequency of Rif^r^ mutants in *P. putida* strains carrying the pheA+C test system at different chromosomal locations

| Strain^a^ | Rif^r^ mutants per 10^9^ cells^b^ |
| --- | --- |
| pheA+C_J | 11.02 (1.87) |
| pheA+C_S | 10.90 (2.22) |
| pheA+C_P | 9.59 (2.11) |
| **pheA+C_O** | **8.90 (1.60)** |
| **pheA+C_G** | **8.65 (2.46)** |
| **pheA+C_B** | **8.50 (2.43)** |

^a^ Strains carrying the mutational target gene opposite to the direction of the movement of replisome in the chromosome are indicated in bold.

^b^Average numbers of Rif^r^ mutants per 1 x 10^9^ cells with 95% confidence intervals are shown. At least 45 independent cultures were examined for each strain.
