# Supplementary material for: Mutation Frequency and Spectrum of Mutations Vary at Different Chromosomal Positions of Pseudomonas putida
Source: PLoS One. 2012 Oct 31;7(10):e48511. doi: 10.1371/journal.pone.0048511 (PMC3485313; doi:10.1371/journal.pone.0048511)
Supplement: Table S7 — Location of the Ptac-pheA+C test system in P. putida PaW85 chromosome. (DOC) [file pone.0048511.s009.doc]

**Table S7. Location of the Ptac-pheA+C test system in *P. putida* PaW85 chromosome**

| Strain | Locus ID | Gene name | Protein name | Insertion position along  the genome^a^ |
| --- | --- | --- | --- | --- |
| Ptac-pheA+C_2 | PP1517 |  | Acriflavin resistance protein | 1,721364 → |
| Ptac-pheA+C_3 | PP2094 | *nasS* | Nitrate binding protein | 2,385886 ← |
| Ptac-pheA+C_4 | PP4517 |  | Conserved hypothetical protein | 5,131645 → |
| Ptac-pheA+C_13 | PP3007 |  | Conserved hypothetical protein | 3,400670 → |

^a^Arrow shows the orientation of the mutational target gene *pheA* along the genome.
