# Supplementary material for: Mutation Frequency and Spectrum of Mutations Vary at Different Chromosomal Positions of Pseudomonas putida
Source: PLoS One. 2012 Oct 31;7(10):e48511. doi: 10.1371/journal.pone.0048511 (PMC3485313; doi:10.1371/journal.pone.0048511)
Supplement: Table S8 — Comparison of mutational spectra in strains carrying the phe-lacI test system at different chromosomal locations. (DOC) [file pone.0048511.s010.doc]

**Table S8.** Comparison of mutational spectra in strains carrying the phe-lacI test system at different chromosomal locations^a^

| Day | Strain | Strain | | | | |
| --- | --- | --- | --- | --- | --- | --- |
|  |  | 105 | 110 | 115 | 117 | 18 |
| Total^b^  (days 3-7) | 110 | *P* < 0.0001 |  |  |  |  |
|  | 115 | *P* < 0.0001 | *P* < 0.0001 |  |  |  |
|  | 117 | *P* < 0.0001 | *P* < 0.0001 | *P* < 0.0001 |  |  |
|  | 18 | *P* < 0.0001 | 0.0011 | *P* < 0.0001 | *P* < 0.0001 |  |
|  | 31 | *P* < 0.0001 | *P* < 0.0001 | 0.0087 | *P* < 0.0001 | *P* < 0.0001 |
| 3-4^c^ | 110 | *P* < 0.0001 |  |  |  |  |
|  | 115 | *P* < 0.0001 | *P* < 0.0001 |  |  |  |
|  | 117 | *P* < 0.0001 | *P* < 0.0001 | *P* < 0.0001 |  |  |
|  | 18 | 0.0001 | 0.0006 | *P* < 0.0001 | *P* < 0.0001 |  |
|  | 31 | *P* < 0.0001 | *P* < 0.0001 | 0.0144 | *P* < 0.0001 | *P* < 0.0001 |
| 5^d^ | 110 | 0.0003 |  |  |  |  |
|  | 115 | *P* < 0.0001 | *P* < 0.0001 |  |  |  |
|  | 117 | *P* < 0.0001 | 0.35 | *P* < 0.0001 |  |  |
|  | 18 | 0.0008 | 0.0694 | *P* < 0.0001 | 0.0049 |  |
|  | 31 | *P* < 0.0001 | *P* < 0.0001 | 0.0659 | *P* < 0.0001 | *P* < 0.0001 |
| 6-7^e^ | 110 | 0.0001 |  |  |  |  |
|  | 115 | *P* < 0.0001 | 0.106 |  |  |  |
|  | 117 | 0.0251 | 0.0008 | *P* < 0.0001 |  |  |
|  | 18 | *P* < 0.0001 | 0.099 | 0.0006 | *P* < 0.0001 |  |
|  | 31 | *P* < 0.0001 | 0.0056 | 0.167 | *P* < 0.0001 | 0.0053 |

^a^The Monte Carlo method according to Adams and Skopek was used. The results are based on 30000 iterations. Spectra are different in a pairwise comparison if P-value is less than 0.0033, since a Bonferroni correction for multiple comparisons α=0.05/15 is used.

^b^All mutations that accumulated for day 7 are taken into comparison of spectra of mutations.

^c^All mutations that accumulated on day 3 and 4 are taken into comparison.

^d^All mutations that accumulated on day 5 are taken into comparison.

^e^All mutations that accumulated on day 6 and 7 are taken into comparison.
