# Supplementary material for: Mutation Frequency and Spectrum of Mutations Vary at Different Chromosomal Positions of Pseudomonas putida
Source: PLoS One. 2012 Oct 31;7(10):e48511. doi: 10.1371/journal.pone.0048511 (PMC3485313; doi:10.1371/journal.pone.0048511)
Supplement: Table S10 — Spectrum of Phe+ mutations on separate days in P. putida strains carrying the phe-lacI test system in various chromosomal positions. (DOC) [file pone.0048511.s012.doc]

Table S10. Spectrum of Phe^+^ mutations on separate days in *P. putida* strains carrying the phe-lacI test system in various chromosomal positions.

**Mutations on days 3 and 4^a^**

| **Position** | **Mutation** | **phe-lacI strains** | | | | | |
| --- | --- | --- | --- | --- | --- | --- | --- |
|  |  | **105** | **110** | **115** | **117** | **18** | **31** |
| -352 | C → T | 0 | 0 | 0 | 0 | 3 (0.406) | 0 |
| 26 | T → C | 0 | 0 | 0 | 0 | 1 (0.135) | 0 |
| 37 | Del G | 0 | 1 (0.422) | 0 | 0 | 0 | 0 |
| 49 | Del T | 0 | 0 | 0 | 0 | 2 (0.27) | 0 |
| 51 | Ins A | 0 | 0 | 0 | 0 | 1 (0.135) | 0 |
| 52 | Ins T | 0 | 0 | 0 | 0 | 1 (0.135) | 0 |
| 53 | A → C | 0 | 1 (0.422) | 0 | 0 | 0 | 0 |
| 53 | A → G | 0 | 1 (0.422) | 0 | 0 | 0 | 0 |
| 54 | G → T | 0 | 0 | 1 (1.45) | 0 | 0 | 0 |
| 55 | A → G | 0 | 1 (0.422) | 0 | 0 | 2 (0.27) | 0 |
| 62 | C → T | 1 (0.142) | 0 | 0 | 2 (1.588) | 1 (0.244) | 0 |
| 89 | T → C | 0 | 1 (0.422) | 0 | 0 | 0 | 0 |
| 96 | Ins 2 nt (CG) | 0 | 0 | 0 | 2 (1.588) | 0 | 0 |
| 149 | Ins T | 0 | 0 | 0 | 0 | 1 (0.135) | 0 |
| 167 | T → A | 0 | 0 | 0 | 0 | 4 (0.541) | 0 |
| 170 | C → T | 0 | 0 | 1 (1.45) | 1 (0.794) | 0 | 0 |
| 221 | Del A | 0 | 0 | 0 | 19 (15.09) | 0 | 0 |
| 232 | G → T | 0 | 0 | 0 | 1 (0.794) | 0 | 0 |
| 253 | Del T | 0 | 0 | 0 | 0 | 1 (0.135) | 0 |
| 391 | C → T | 0 | 1 (0.422) | 0 | 0 | 0 | 0 |
| 403 | Ins T | 0 | 0 | 0 | 1 (0.794) | 0 | 0 |
| 405 | Ins T | 1 (0.142) | 0 | 0 | 0 | 0 | 0 |
| 409 | Ins G | 1 (0.142) | 0 | 0 | 0 | 0 | 0 |
| 457 | C → T | 0 | 1 (0.422) | 0 | 0 | 0 | 0 |
| 475 | Ins T | 0 | 0 | 0 | 1 (0.794) | 0 | 0 |
| 476 | Ins T | 1 (0.142) | 0 | 0 | 2 (1.588) | 0 | 0 |
| 490 | Ins A | 0 | 0 | 0 | 0 | 1 (0.135) | 0 |
| 504 | Ins G | 0 | 0 | 0 | 0 | 0 | 3 (2.47) |
| 552 | Del G | 1 (0.142) | 0 | 0 | 0 | 0 | 0 |
| 592 | Ins CTGG | 1 (0.142) | 0 | 34 (49.3) | 1 (0.794) | 3 (0.406) | 42 (34.57) |
| 592 | Del CTGG | 12 (1.708) | 38 (16.04) | 2 (2.9) | 12 (9.528) | 30 (4.056) | 4 (3.293) |
| 603 | G → A | 0 | 0 | 0 | 0 | 0 | 1 (0.823) |
| 669 | Del G | 0 | 0 | 0 | 1 (0.794) | 0 | 0 |
| 705 | Ins T | 0 | 0 | 0 | 1 (0.794) | 0 | 0 |
| 754 | G → T | 8 (1.138) | 0 | 0 | 0 | 0 | 0 |
| 858 | A → C | 0 | 0 | 0 | 1 (0.794) | 0 | 0 |
| 916 | C → T | 0 | 2 (0.844) | 0 | 0 | 0 | 0 |
| IS1411 |  | 0 | 3 (1.266) | 5 (7.25) | 3 (2.382) | 0 | 0 |
| unknown |  | 0 | 0 | 1 (1.45) | 2 (1.588) | 0 | 0 |
| Overall mutation frequency | | 3.7 | 21.1 | 63.8 | 39.7 | 6.76 | 41.16 |
| Total number analyzed | | 26 | 50 | 44 | 50 | 50 | 50 |

^a^Frequency of mutation per site calculated per total number of Phe^+^ mutants accumulated per 1 x 10^7^cells during days 3-4 is shown in parentheses.

**Mutations on day 5^a^**

| **Position** | **Mutation** | **phe-lacI strains** | | | | | |
| --- | --- | --- | --- | --- | --- | --- | --- |
|  |  | **105** | **110** | **115** | **117** | **18** | **31** |
| -354 | Del T | 1 (0.106) | 0 | 0 | 0 | 0 | 0 |
| -353 | Del 2 nt (TC) | 1 (0.106) | 0 | 0 | 0 | 0 | 0 |
| -352 | C → T | 0 | 9 (5.026) | 4 (2.963) | 11 (17.57) | 5 (0.988) | 2 (0.919) |
| -352 | C → G | 1 (0.106) | 0 | 0 | 0 | 0 | 0 |
| -351 | G → T | 1 (0.106) | 0 | 0 | 0 | 0 | 1 (0.46) |
| -351 | G → A | 1 (0.106) | 0 | 0 | 1 (1.597) | 0 | 0 |
| -347 | A → G | 0 | 0 | 1 (0.741) | 0 | 0 | 0 |
| -347 | Ins A | 0 | 0 | 0 | 3 (4.792) | 0 | 0 |
| -345 | Ins C | 0 | 0 | 0 | 1 (1.597) | 0 | 0 |
| -3 | Del 288 nt | 1 (0.106) | 0 | 0 | 0 | 0 | 0 |
| 13 | A → G | 0 | 0 | 0 | 0 | 1 (0.198) | 0 |
| 44 | T → C | 0 | 0 | 0 | 0 | 0 | 4 (1.839) |
| 51 | Ins A | 0 | 0 | 0 | 0 | 1 (0.198) | 0 |
| 53 | A → G | 0 | 0 | 0 | 1 (1.597) | 0 | 0 |
| 55 | A → G | 0 | 2 (1.117) | 0 | 1 (1.597) | 1 (0.198) | 0 |
| 62 | C → T | 2 (0.213) | 0 | 0 | 0 | 1 (0.198) | 0 |
| 100 | Ins 10 nt | 0 | 0 | 0 | 1 (1.597) | 0 | 0 |
| 111 | Ins 10 nt | 1 (0.106) | 0 | 0 | 0 | 3 (0.593) | 0 |
| 131 | G → C | 1 (0.106) | 0 | 0 | 0 | 0 | 0 |
| 140 | A → G | 0 | 1 (0.558) | 0 | 0 | 0 |  |
| 158 | C → A | 0 | 1 (0.558) | 0 | 0 | 0 | 0 |
| 178 | C → T | 4 (0.425) | 0 | 0 | 0 | 0 | 0 |
| 299 | Del 149 nt | 0 | 1 (0.558) | 0 | 0 | 0 | 0 |
| 300 | Del 10 nt | 0 | 0 | 0 | 0 | 7 (1.384) | 0 |
| 310 | Del G | 0 | 0 | 1 (0.741) | 0 | 0 | 0 |
| 394 | Del 66 nt | 1 (0.106) | 0 | 0 | 0 | 0 | 0 |
| 399 | Ins C | 0 | 0 | 0 | 1 (1.597) | 0 | 0 |
| 479 | Ins T | 1 (0.106) | 0 | 0 | 0 | 0 | 0 |
| 592 | Ins CTGG | 0 | 1 (0.558) | 15 (11.11) | 0 | 2 (0.395) | 15 (6.895) |
| 592 | Del CTGG | 4 (0.425) | 4 (2.234) | 0 | 3 (4.792) | 4 (0.791) | 4 (1.839) |
| 669 | Del G | 0 | 0 | 0 | 1 (1.234) | 1 (0.198) | 0 |
| 751 | C → A | 0 | 0 | 0 | 0 | 0 | 1 (0.46) |
| 754 | G → T | 2 (0.213) | 0 | 0 | 0 | 0 | 0 |
| 779 | Ins 2 nt (CA) | 0 | 0 | 1 (0.741) | 0 | 0 | 0 |
| 780 | C → A | 1 (0.106) | 0 | 0 | 0 | 0 | 0 |
| 983 | C → T | 0 | 0 | 0 | 1 (1.597) | 0 | 0 |
| IS1411 |  | 8 (0.85) | 5 (2.792) | 1 (0.741) | 2 (3.195) | 4 (0.791) | 2 (0.919) |
| unknown |  | 1 (0.106) | 1 (0.558) | 1 (0.741) | 1 (1.597) | 0 | 1 (0.46) |
| Overall mutation frequency | | 3.4 | 13.96 | 17.78 | 43.13 | 5.93 | 13.79 |
| Total number analyzed | | 32 | 25 | 24 | 27 | 30 | 30 |

^a^Frequency of mutation per site calculated per total number of Phe^+^ mutants accumulated per 1 x 10^7^cells per day 5 is shown in parentheses.

**Mutations on days 6-7^a^**

| **Position** | **Mutation** | **phe-lacI strains** | | | | | |
| --- | --- | --- | --- | --- | --- | --- | --- |
|  |  | **105** | **110** | **115** | **117** | **18** | **31** |
| -353 | Del 2 nt (TC) | 0 | 0 | 0 | 1 (1.477) | 0 | 0 |
| -352 | C → T | 14 (19.09) | 0 | 2 (2.034) | 5 (7.386) | 0 | 1 (1.174) |
| -352 | C → G | 1 (1.363) | 0 | 0 | 0 | 0 | 0 |
| -351 | G → A | 12 (16.36) | 26 (48.69) | 27 (27.45) | 15 (22.16) | 38 (14.48) | 38 (44.6) |
| -349 | T → G | 0 | 0 | 0 | 0 | 1 (0.381) | 0 |
| -349 | T → C | 0 | 0 | 2 (2.034) | 0 | 0 | 0 |
| -348 | C → A | 0 | 0 | 1 (1.017) | 0 | 0 | 0 |
| -347 | Ins A | 0 | 1 (1.873) | 0 | 0 | 0 | 0 |
| -346 | C → T | 0 | 1 (1.873) | 0 | 1 (1.477) | 0 | 0 |
| 22 | Ins 22 nt | 1 (1.363) | 0 | 0 | 0 | 0 | 0 |
| 24 | T → C | 1 (1.363) | 0 | 0 | 0 | 0 | 0 |
| 29 | C → T | 0 | 0 | 0 | 1 (1.477) | 0 | 0 |
| 55 | A → G | 0 | 0 | 0 | 3 (4.432) | 1 (0.381) | 0 |
| 75 | C → G | 1 (1.363) | 0 | 0 | 0 | 0 | 0 |
| 140 | A → G | 0 | 0 | 0 | 0 | 0 | 1 (1.174) |
| 141 | C → G | 1 (1.363) | 0 | 0 | 0 | 0 | 1 (1.174)) |
| 146 | C → T | 1 (1.363) | 0 | 0 | 0 | 1 (0.381) | 0 |
| 178 | C → T | 0 | 0 | 0 | 5 (7.386) | 0 | 0 |
| 253 | Del 14 nt | 0 | 0 | 0 | 1 (1.477) | 0 | 0 |
| 291 | G → T | 2 (2.727) | 0 | 0 | 0 | 0 | 0 |
| 313 | G → C | 0 | 1 (1.873) | 0 | 0 | 0 | 0 |
| 313 | G → T | 1 (1.363) | 0 | 0 | 0 | 0 | 0 |
| 458 | Ins C | 0 | 0 | 1 (1.017) | 0 | 0 | 0 |
| 460 | Ins G | 0 | 0 | 1 (1.017) | 0 | 0 | 0 |
| 541 | C → T | 0 | 0 | 0 | 0 | 2 (0.762) | 0 |
| 592 | Ins CTGG | 2 (2.727) | 2 (3.745) | 9 (9.151) | 0 | 1 (0.381) | 8 (9.39) |
| 592 | Del CTGG | 2 (2.727) | 2 (3.745) | 1 (1.017) | 3 (4.432) | 3 (1.143) | 0 |
| 653 | G → T | 1 (1.363) | 0 | 0 | 1 (1.477) | 0 | 0 |
| 661 | Ins C | 1 (1.363) | 0 | 0 | 1 (1.477) | 0 | 0 |
| 682 | C → T | 0 | 0 | 0 | 0 | 1 (0.381) | 0 |
| IS1411 |  | 9 (12.27) | 4 (7.491) | 2 (2.034) | 11 (16.25) | 4 (0.975) | 0 |
| unknown |  | 2 (2.727) | 0 | 1 (1.017) | 2 (2.954) | 2 (0.762) | 1 (1.174) |
| Overall mutation frequency | | 70.9 | 69.29 | 47.79 | 73.86 | 19.05 | 58.69 |
| Total number analyzed | | 52 | 37 | 47 | 50 | 50 | 50 |

^a^Frequency of mutation per site calculated per total number of Phe^+^ mutants accumulated per 1 x 10^7^cells during days 6-7 is shown in parentheses.
