# Supplementary material for: Mutation Frequency and Spectrum of Mutations Vary at Different Chromosomal Positions of Pseudomonas putida
Source: PLoS One. 2012 Oct 31;7(10):e48511. doi: 10.1371/journal.pone.0048511 (PMC3485313; doi:10.1371/journal.pone.0048511)
Supplement: Table S11 — Comparison of mutational spectra on different time periods in strains carrying the phe-lacI test system at various chromosomal locations. (DOC) [file pone.0048511.s013.doc]

**Table S11.** Comparison of mutational spectra on different time periods in strains carrying the phe-lacI test system at various chromosomal locations^a^

| Comparison | 105 | 110 | 115 | 117 | 18 | 31 |
| --- | --- | --- | --- | --- | --- | --- |
| Days 3-4 versus day 5 | 0.008 | 0.0099 | 0.28 | 0.0002 | *P* < 0.0001 | 0.0039 |
| Days 3-4 versus days 6-7 | 0.0003 | 0.028 | 0.331 | *P* < 0.0001 | 0.0622 | 0.0836 |
| Day 5 versus  days 6-7 | 0.0084 | 0.714 | 0.307 | 0.0458 | 0.0991 | 0.0836 |

^a^ The Monte Carlo method according to Adams and Skopek was used. The results are based on 30 000 iterations. Spectra are different in a pairwise comparison if P-value is less than 0.0167, since a Bonferroni correction for multiple comparisons α=0.05/3 is used.
