# Supplementary material for: Mutation Frequency and Spectrum of Mutations Vary at Different Chromosomal Positions of Pseudomonas putida
Source: PLoS One. 2012 Oct 31;7(10):e48511. doi: 10.1371/journal.pone.0048511 (PMC3485313; doi:10.1371/journal.pone.0048511)
Supplement: Table S12 — The results of pairwise comparison of mutation frequency on different time periods at individual sites obtained with chi-square test. Only the results with P <0.005 are shown. (DOC) [file pone.0048511.s014.doc]

**Table S12.** The results of pairwise comparison of mutation frequency on different time periods at individual sites obtained with chi-square test. Only the results with *P* < 0.05 are shown.

Days 3-4

|  | **phe-lacI strains** | ***P*-value** |
| --- | --- | --- |
| **Single nt mutation in *lacI* gene** |  |  |
| **221 del A** | 117 vs. 105 | <0.001 |
|  | 117 vs. 110 | <0.001 |
|  | 117 vs. 115 | <0.001 |
|  | 117 vs. 18 | <0.001 |
|  | 117 vs. 31 | <0.001 |
| **754 G→T** | 105 vs. 110 | <0.001 |
|  | 105 vs. 115 | 0.001 |
|  | 105 vs. 117 | <0.001 |
|  | 105 vs. 18 | <0.001 |
|  | 105 vs. 31 | <0.001 |
| **592 ins CTGG** |  |  |
|  | 105 vs. 115 | <0.001 |
|  | 105 vs. 31 | <0.001 |
|  | 110 vs. 115 | <0.001 |
|  | 110 vs. 31 | <0.001 |
|  | 115 vs. 117 | <0.001 |
|  | 115 vs. 18 | <0.001 |
|  | 117 vs. 31 | <0.001 |
|  | 18 vs. 31 | <0.001 |
| **592 del CTGG** |  |  |
|  | 105 vs. 110 | 0.009 |
|  | 105 vs. 115 | 0.001 |
|  | 105 vs. 117 | 0.049 |
|  | 105 vs. 31 | <0.001 |
|  | 110 vs. 115 | <0.001 |
|  | 110 vs. 117 | <0.001 |
|  | 110 vs. 31 | <0.001 |
|  | 115 vs. 117 | 0.008 |
|  | 115 vs. 18 | <0.001 |
|  | 117 vs. 18 | <0.001 |
|  | 117 vs. 31 | 0.029 |
|  | 18 vs. 31 | <0.001 |
| **Insertion of IS*1411*** |  |  |
|  | 115 vs. 18 | 0.014 |
|  | 115 vs. 31 | 0.014 |

Day 5

|  | **phe-lacI strains** | ***P*-value** |
| --- | --- | --- |
| **Operator mutations** |  |  |
| **−352 C→T** | 105 vs. 110 | <0.001 |
|  | 105 vs. 115 | 0.017 |
|  | 105 vs. 117 | <0.001 |
|  | 105 vs. 18 | 0.016 |
|  | 110 vs. 31 | 0.007 |
|  | 117 vs. 18 | 0.043 |
|  | 117 vs. 31 | 0.002 |
| **Large deletions** |  |  |
| **300 del 10 nt** | 18 vs. 105 | 0.004 |
|  | 18 vs. 110 | 0.010 |
|  | 18 vs. 115 | 0.011 |
|  | 18 vs. 117 | 0.007 |
|  | 18 vs. 31 | 0.005 |
| **592 ins CTGG** |  |  |
|  | 105 vs. 115 | <0.001 |
|  | 105 vs. 31 | <0.001 |
|  | 110 vs. 115 | <0.001 |
|  | 110 vs. 31 | <0.001 |
|  | 115 vs. 117 | <0.001 |
|  | 115 vs. 18 | <0.001 |
|  | 117 vs. 31 | <0.001 |
|  | 18 vs. 31 | <0.001 |
| **592 del CTGG** |  |  |
|  | 110 vs. 115 | 0.041 |
| **Insertion of IS*1411*** |  |  |
|  | 105 vs. 115 | 0.036 |

Days 6-7

|  | **phe-lacI strains** | ***P*-value** |
| --- | --- | --- |
| **Operator mutations** |  |  |
| **−352 C→T** | 105 vs. 110 |  |
|  | 105 vs. 115 | 0.048 |
|  | 105 vs. 31 | 0.002 |
|  |  |  |
|  | 110 vs. 31 | 0.041 |
|  | 115 vs. 117 | 0.046 |
|  | 117 vs. 31 | 0.002 |
| **−351 G→A** | 105 vs. 110 | 0.026 |
|  | 105 vs. 115 | 0.022 |
|  | 105 vs. 18 | 0.001 |
|  | 105 vs. 31 | 0.001 |
|  | 110 vs. 117 | 0.031 |
|  | 115 vs. 117 | 0.027 |
|  | 117 vs. 18 | 0.001 |
|  | 117 vs. 31 | 0.001 |
| **Single nt mutation in *lacI* gene** |  |  |
| **221 del A** | 117 vs. 105 | <0.001 |
|  | 117 vs. 110 | <0.001 |
|  | 117 vs. 115 | <0.001 |
|  | 117 vs. 18 | <0.001 |
|  | 117 vs. 31 | <0.001 |
| **754 G→T** | 105 vs. 110 | 0.001 |
|  | 105 vs. 115 | 0.001 |
|  | 105 vs. 117 | 0.001 |
|  | 105 vs. 18 | <0.001 |
|  | 105 vs. 31 | <0.001 |
| **592 ins CTGG** |  |  |
|  | 105 vs. 115 | <0.001 |
|  | 105 vs. 31 | <0.001 |
|  | 110 vs. 115 | <0.001 |
|  | 110 vs. 31 | <0.001 |
|  | 115 vs. 117 | <0.001 |
|  | 115 vs. 18 | <0.001 |
|  | 117 vs. 31 | <0.001 |
|  | 18 vs. 31 | <0.001 |
| **592 del CTGG** |  |  |
|  | 105 vs. 110 | <0.001 |
|  | 105 vs. 115 | <0.001 |
|  | 105 vs. 18 | 0.026 |
|  | 105 vs. 31 | 0.011 |
|  | 110 vs. 115 | <0.001 |
|  | 110 vs. 117 | <0.001 |
|  | 110 vs. 31 | 0.001 |
|  | 115 vs. 117 | 0.001 |
|  | 115 vs. 18 | <0.001 |
|  | 117 vs. 18 | 0.005 |
|  | 117 vs. 31 | 0.033 |
|  | 18 vs. 31 | <0.001 |
| **Insertion of IS*1411*** |  |  |
|  | 105 vs. 115 | 0.043 |
|  | 105 vs. 18 | 0.001 |
|  | 105 vs. 31 | <0.001 |
|  | 110 vs. 18 | 0.017 |
|  | 110 vs. 31 | 0.002 |
|  | 115 vs. 31 | 0.032 |
|  | 117 vs. 18 | 0.004 |
|  | 117 vs. 31 | 0.001 |
